# mTOR regulates GPVI-mediated platelet activation

**DOI:** 10.1186/s12967-021-02756-y

**Published:** 2021-05-10

**Authors:** Longsheng Wang, Gang Liu, Nannan Wu, Baiyun Dai, Shuang Han, Qiaoyun Liu, Fang Huang, Zhihua Chen, Weihong Xu, Dajing Xia, Cunji Gao

**Affiliations:** 1 Chronic Disease Research Institute, Department of Nutrition and Food Hygiene, Zhejiang University School of Public Health, 866 Yu-Hang-Tang Road, Hangzhou, 310058 China; 2 Department of Toxicology, Zhejiang University School of Public Health, 866 Yu-Hang-Tang Road, Hangzhou, 310058 China; 3 Department of Respiratory Medicine, Second Affiliated Hospital, Zhejiang University School of Medicine, 88 Jiefang Road, Hangzhou, 310009 China; 4 Zhejiang Hospital, 12 Lingyin Road, Hangzhou, 310013 China; 5 Department of Pharmacology, School of Basic Medical Sciences, Guizhou Medical University, Guiyang, Guizhou China; 6 Blood Research Institute, Blood Center of Wisconsin, Milwaukee, Milwaukee, WI 53201 USA

**Keywords:** Platelets, mTOR, GPVI, Dense granule secretion (ATP release), Pkcδ

## Abstract

**Background:**

Due to mTOR (mammalian/mechanistic target of rapamycin) gene-loss mice die during embryonic development, the role of mTOR in platelets has not been evaluated using gene knockout technology.

**Methods:**

A mouse model with megakaryocyte/platelet-specific deletion of mTOR was established, and be used to evaluate the role of mTOR in platelet activation and thrombus formation.

**Results:**

mTOR^−/−^ platelets were deficient in thrombus formation when grown on low-concentration collagen-coated surfaces; however, no deficiency in thrombus formation was observed when mTOR^−/−^ platelets were perfused on higher concentration collagen-coated surfaces. In FeCl_3_-induced mouse mesenteric arteriole thrombosis models, wild-type (WT) and mTOR^*−/−*^ mice displayed significantly different responses to low-extent injury with respect to the ratio of occluded mice, especially within the first 40 min. Additionally, mTOR^−/−^ platelets displayed reduced aggregation and dense granule secretion (ATP release) in response to low doses of the glycoprotein VI (GPVI) agonist collagen related peptide (CRP) and the protease-activated receptor-4 (PAR4) agonist GYPGKF-NH_2_; these deficiencies were overcame by stimulation with higher concentration agonists, suggesting dose dependence of the response. At low doses of GPVI or PAR agonist, the activation of α_IIb_β_3_ in mTOR^*−/−*^ platelets was reduced. Moreover, stimulation of mTOR^−/−^ platelets with low-dose CRP attenuated the phosphorylation of S6K1, S6 and Akt Ser473, and increased the phosphorylation of PKCδ Thr505 and PKCε Ser729. Using isoform-specific inhibitors of PKCs (δ, ɛ, and α/β), we established that PKCδ/ɛ, and especially PKCδ but not PKCα/β or PKCθ, may be involved in low-dose GPVI-mediated/mTOR-dependent signaling.

**Conclusion:**

These observations indicate that mTOR plays an important role in GPVI-dependent platelet activation and thrombus formation.

## Background and introduction

At the site of an injured vessel, platelets are recruited and undergo rapid aggregation to form a hemostatic or thrombotic plug. After recruitment, subendothelial matrix collagen interacts with platelet surface glycoprotein VI (GPVI) and stimulates the platelets at the site of recruitment [1]. The process of platelet activation is complex and involves several pathways and signaling molecules, and there are a limited number of studies that directly demonstrate platelet-specific functions of key modulators using gene knockout technology [2].

Mammalian target of rapamycin (mTOR), also called mechanistic target of rapamycin, is a serine/threonine kinase that is expressed in megakaryocytes, pro-platelets, and circulating platelets [3]. mTOR interacts with other proteins and assembles into two complexes: mTOR complex1 (mTORC1) and mTOR complex2 (mTORC2). The upstream and downstream signals of these two complexes, as well as their sensitivities to rapamycin, are different. Rapamycin inhibits mTORC1, but not mTORC2, during acute treatment [4]; however, in some cell types, mTORC2 signaling is reduced by long-term treatment with rapamycin [5, 6]. Based on its inhibition of these complexes, rapamycin is used as an anti-fungal agent, immune-suppressive agent and anti-tumor agent. Some new inhibitors of mTOR, such as Torin2 and NVP-BEZ235, have also been developed for the inhibition of mTORC1 and mTORC2 [7, 8]. Evidence using these inhibitors suggests that rapamycin affects platelet aggregation, dense granule secretion, spreading on fibrinogen and clot retraction, and thrombus formation [9–13]. However, because mTOR gene-deletion mice die during early preimplantation [14], the role of mTOR in platelets has not been evaluated using gene knockout technology.

The mTORC1 and mTORC2 complexes have been shown to phosphorylate and activate a variety of downstream signaling mediators, including S6K1, S6, and Akt [4, 15, 16]. Additionally, mTOR interacts with mitogen-activated protein kinases, including Erk, in various cell types[17, 18] PKCs have been reported to be regulated by mTOR in various cell types [4] and to mediate platelet aggregation, dense- and alpha-granule secretion, and α_IIb_β_3_ activation and spreading on fibrinogen [9, 10, 13, 19–34]. The expression of cPKCα, cPKCβ, nPKCδ and nPKCθ in human platelets has been well established [33, 35, 36], though the expression of PKCη in human platelets has been disputed [28, 33, 35–37]. Furthermore, PKCε is expressed in human megakaryocytes, but not in human platelets [28, 36, 38], while mouse platelets express PKCε [28, 39]. The phosphorylation of Thr497 [27] in the activation loop of PKCα, Thr505 [35] in the activation loop of PKCδ, and Thr538 [40, 41] in the activation loop of PKCθ, is thought to be critical for their activity [42], and the phosphorylation of PKCβ Thr641, PKCβ Ser660 [27], and PKCε Ser729 [20], has been shown to be essential for activation [42].

In this study, we generated a mouse model with megakaryocyte/platelet-specific deletion of the mTOR gene. Our results demonstrate that mTOR plays positive roles in thrombus formation in vitro when perfused on low-concentration collagen-coated surfaces and in vivo in response to low-extent FeCl_3_-induced injury. The regulation of thrombus formation in vitro was found to be dependent on the collagen concentration and dependent on the extent of FeCl_3_-induced injury in vivo. We also demonstrate that mTOR plays a positive role in low-dose GPVI-mediated platelet activation. These findings provide direct evidence for a pivotal role of mTOR in platelets.

## Materials and methods

### Reagents

Thrombin, ADP, apyrase, PGE1, and rapamycin were from Sigma-Aldrich. Collagen and luciferase were from Chrono-Log Corp. Human fibrinogen was from Enzyme Research Laboratories (South Bend, IN, USA). APC-conjugated fibrinogen, GYPGKF-NH_2_ (a Protease-activated receptor-4 (PAR4) agonist peptide) and synthetic collagen-related peptide (CRP) [43] were kindly donated by Peter J. Newman (Blood Center of Wisconsin; Milwaukee, WI, USA). PE-conjugated anti-mouse CD41 (α_II_b), FITC-conjugated anti-mouse CD62 (P-selectin), and antibodies to PKCδ, PKCβ, PKCθ, and PKCɛ were from BD Biosciences. FITC-conjugated anti-mouse GPVI (JAQ1), FITC-conjugated anti-mouse CD42b (Xia. B2), and PE-labeled JON/A antibodies were from Emfret Analytics (Eibelstadt, Germany). Antibodies to mTOR, Rictor (D16H9), phospho-Akt Ser473, β-actin (SH10D10), S6K1, phospho-PKC Substrate Motif [(R/KXpSX(R/K)] (be usually thought to recognize substrates of cPKCs), and phospho-Lyn Tyr507 were from Cell Signaling Technology. Torin1 and antibodies to phospho-PKCδ Thr505, S6, phospho-S6 Ser235/236, phospho-Erk Thr202/Tyr204, and Erk were kindly provided by Hanming Shen (professor at the National University of Singapore). Antibodies to Raptor, phospho-PKCα Thr497, PKCα, and phospho-PKCβ Ser660, were obtained from Abcam. Antibodies to phospho-S6K1 Thr389, phospho-PKCɛ Ser729, and phospho-PKCβ Thr641, were from Santa Cruz Biotechnology. Antibodies to phospho-PKCθ Thr538 were kindly provided by Hanming Shen/purchased from Santa Cruz Biotechnology. Rottlerin was from Merck Biosciences (Beeston, United Kingdom). Go 6976 was from Biomol (Enzo Life Sciences). Calcein was from Invitrogen (Eugene, OR, USA). Fluorescein-labeled phalloidin was from Molecular Probes (Eugene, OR, USA). Other reagents were of analytical grade. The δV1-1 (selective PKCδ peptide inhibitor, SFNSYELGSL) [21, 44], ɛV1–2 (selective PKCɛ peptide inhibitor, EAV SLK PT) [45–48], were synthesized and purified (≥ 98%) by China Peptides Co., Ltd. (Shanghai, China). The peptides were conjugated to the Tat-carrier peptide (YGR KKR RQR RR) [49] via a cysteine-cysteine S–S bond at its N terminus.

### Mice

mTOR^fl/fl^ mice [50] in a C57BL/6 genetic background were mated with PF4-Cre + mice [51] to obtain mTOR^fl/wt^PF4-Cre+ mice. Further backcrossing with mTOR^fl/fl^ gave rise to mTOR^fl/fl^PF4-Cre+ (mTOR^−/−^) mice, which have mTOR deficiency in their platelets. PCR was used for genotyping the mice, while western blots were used for confirming the deletion of mTOR in platelets. Genotyping was performed by PCR, using the primer pair PF4-Cre F/PF4-Cre R (predicted 450 bp product in PF4-Cre positive mice) and primer pair mTOR^fl/fl^ F/ mTOR^fl/fl^ R (expected product 349 bp in WT; 349/533 bp in mTOR^fl/wt^; and 533 bp in mTOR^fl/fl^). The sequences of these primers are as follows: PF4-Cre Forward: 5′-CCCATACAGCACACCTTTTG-3′; PF4-Cre Reverse: 5′-TGCACAGTCAGCAGGTT-3′; mTOR^fl/fl^ Forward: 5′-TTATGTTTGATAATTGCA GTTTTGGCTAGCAGT-3′; mTOR^fl/fl^ Reverse: 5′-TTTAGGACTCCTTCTGTGACATAC ATTTCCT-3′. All experiments were performed under the Guide for the Care and Use of Laboratory Animal (The National Academy Press, 2011) and were approved by the Board of Animal Study of Zhejiang University. mTOR^fl/fl^PF4-Cre^+^ (mTOR^−/−^) and PF4-Cre^−^ (Wild-type, WT) littermate mice at the age of 8–12 weeks were used for experiments.

### Assessment of hematologic parameters

Hematologic parameters were determined on ethylenediamine tetraacetic acid samples collected from mice using an automatic cell counter (Sysmex F-820, Kobe, Japan).

### Platelet preparation

Mice were anesthetized by intraperitoneal injection with chloral hydrate. Whole blood was collected from the inferior vena cava and anticoagulated with 1/8 vol ACD (75 mM sodium citrate, 39 mM citric acid, and 135 mM dextrose, pH 6.5). The blood was then diluted 1:1 with Modified Tyrode’s buffer (20 mM HEPES, 137 mM NaCl, 13.8 mM NaHCO3, 2.5 mM KCl, 0.36 mM NaH_2_PO_4_, 5.5 mM glucose, pH 7.4) and centrifuged for 10 min at 180*g*; platelet-rich plasma (PRP) was collected in a fresh tube. Platelet pellets were obtained by centrifugation of the PRP at 700×*g* for 10 min, washing in HEPES-buffered Tyrode containing 1/9 vol ACD and 50 ng/mL PGE1 and resuspension in modified Tyrode’s buffer (20 mM HEPES, 137 mM NaCl, 13.8 mM NaHCO3, 2.5 mM KCl, 0.36 mM NaH_2_PO_4_, 5.5 mM glucose, pH 7.4) as previously described [52].

### Aggregation and dense granule secretion (ATP release)

Platelet aggregation and dense granule secretion (ATP release) experiments were performed as described previously [53]. Briefly, washed platelets and PRP were challenged with the indicated agonists at 37 °C while they were stirred using an aggregometer (ChronoLog; Havertown, PA, USA), and dense granule secretion (ATP release) was measured by ATP release and monitored in parallel with aggregation by the addition of luciferin/luciferase. Washed platelets (2.0 × 10^8^/mL) in modified Tyrode’s buffer were challenged with GYPGKF-NH_2_, collagen, and CRP, while PRP was stimulated with ADP. The platelets were incubated with the indicated inhibitors at a temperature of 37 °C for 10 min before stimulation.

### Flow cytometry

Flow cytometric analysis was performed and developed as described previously [54]. Washed platelets were diluted to 2.0 × 10^7^/mL in modified Tyrode’s buffer with Ca^2+^ and were pre-incubated with either FITC-conjugated CD62P, FITC-conjugated GPVI, PE-conjugated CD41, PE-conjugated JON/A, APC-conjugated fibrinogen, FITC-conjugated CD42b, or isotype control antibodies. In some experiments, agonists were used to stimulate the washed platelets after incubation with antibodies as indicated [54]. The samples were fixed by adding 2% (vol/vol) formaldehyde. Flow cytometry was performed using a FACS Calibur flow cytometer.

### Western blotting

Washed platelets were resuspended and adjusted to 2 × 10^8^/mL in Tyrode's buffer. Then, the platelets were stimulated with the indicated agonists in a Chrono-Log Aggregometer at 37 °C for 5.5 min. Immunoblotting was carried out as described previously[55]. Lysates were prepared and analyzed by SDS-PAGE, electrotransferred to PVDF membranes, blocked with 5% (w/v) BSA in TBST and probed with primary antibodies. The membranes were washed in TBST and incubated with appropriate secondary antibodies. Immunoreactive bands were visualized with enhanced chemiluminescence detection reagents using a Syngene G: BOX Chemi XR system and quantified by Image J.

### In vitro* thrombus formation under flow conditions*

Thrombus formation was assessed and developed as described previously [56] using a BiofluxTM 200 system (Fluxion, South San Francisco, CA). Briefly, Bioflux plates were primed and coated overnight with 20 μg/mL or 50 μg/mL of fibrillar collagen I and then blocked with 0.5% BSA/PBS (W/V) solution for 15 min. Whole blood was labeled with mepacrine at 37 °C for 30 min and then perfused in micro-channels at a shear force of 40 dynes/cm^2^ for 5 min, during which time platelet adhesion and aggregation were monitored by fluorescent microscopy. After perfusion, the adherent platelets were observed with an inverted fluorescence microscope (Nikon Ti-S, Tokyo, Japan). The coverage area of the platelets and the fluorescence intensity were measured using Bioflux software (Fluxion).

### Statistical analysis

The results are expressed as means ± SEM. The paired or unpaired Student *t*-test was used to evaluate the statistical significance of the data.

## Results

### Megakaryocyte- and platelet-specific mTOR-deficient mice display normal hematopoietic parameters but their platelets show impaired thrombus formation when perfused on low-concentration collagen-coated surfaces

**Fig. 1:**
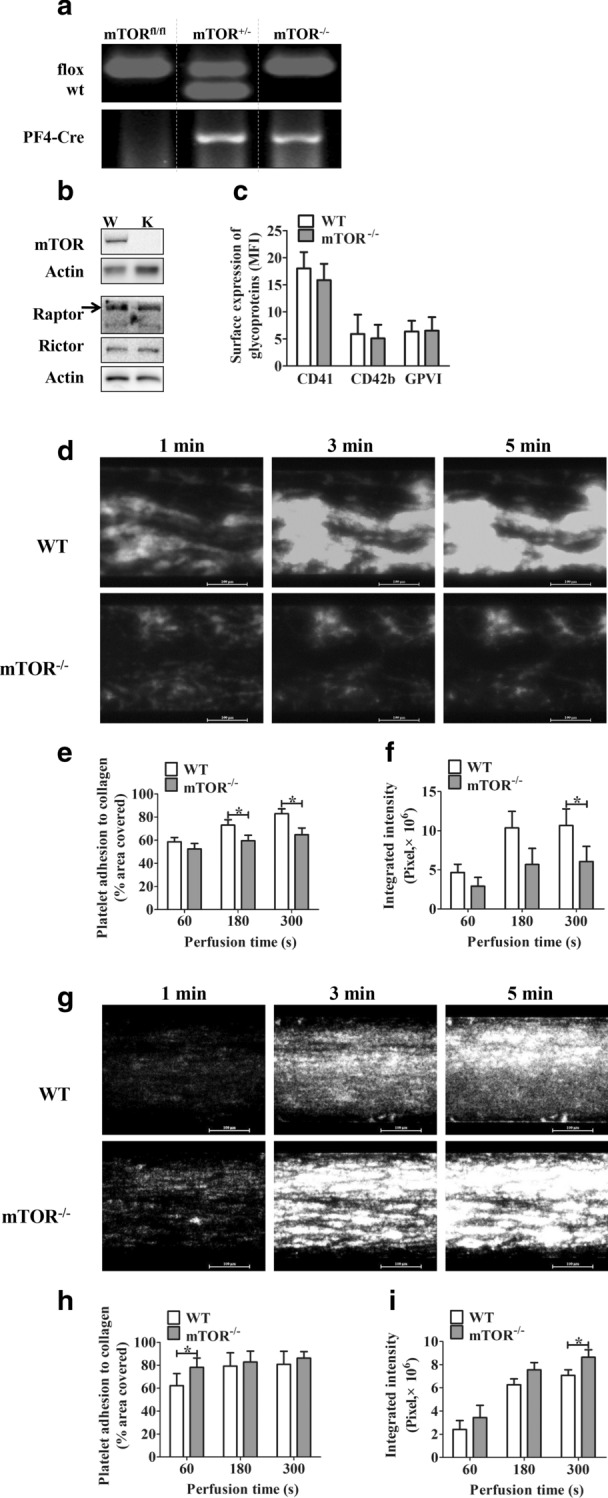
Deletion of mTOR protein in platelets. **a** Genotyping of mTOR^fl/fl^, mTOR^fl/wt^ PF4-Cre + (mTOR^+/−^) and mTOR^fl/fl^ PF4-Cre + (mTOR^−/−^) mice using polymerase chain reaction. The top and bottom images were cropped from different gels and full-length/original gels were shown in Additional file 3: Part III. **b** Lysates from PF4-Cre^−^ (wildtype, WT, W) littermate, or mTOR^−/−^ (KO, K) platelets were immunoblotted with mTOR, Raptor, Rictor, and Actin antibodies, these images (separated by horizontal white space) were cropped from the different/same gels and full-length/original blots were shown in Additional file 3: Part III. **c** Flow cytometric analysis of the surface expression of CD41 (α_IIb_β_3)_, CD42b (GPIbα), and GPVI of resting WT and mTOR^−/−^ murine platelets. Results are expressed as mean fluoresce intensity (MFI) ± SEM (n = 3). **d**–**f** Representative photomicrographs and quantification of the adhesion and aggregation of WT or mTOR^−/−^ platelets on 20 μg/mL or **g**–**i** 50 μg/mL type I fibrillar collagen-coated surface. The fluorescent platelets adhering to coated Bioflux micro flow chambers were recorded by video (exposure time were fixed as 1.5 or 0.8 s, respectively). The coverage surface area and total integrated fluorescence were quantified by Bioflux software (Fluxion) as a measure of platelet thrombi formation. Quantified results are expressed as the mean percentage of surface coverage (left panels) or mean integrated fluorescence intensity (right panels) ± SEM (n ≥ 3 per group, * indicates P < 0.05, paired Student’s *t* test)

mTOR gene knockout embryos of mice are severely runted and die during early preimplantation [14]. Therefore, we used Cre recombinase–mediated excision to delete mTOR specifically in megakaryocytes and platelets. To confirm platelet-specific gene deletion, we used polymerase chain reaction to genotype mTOR^fl/fl^, mTOR^fl/wt^PF4-Cre+ (mTOR^+/−^) and mTOR^fl/fl^ PF4-Cre+ (mTOR^−/−^) mice (Fig. 1a). Western blotting confirmed mTOR specifically-deficient in platelets (Fig. 1b). The surface expression of platelet glycoproteins CD41 (α_IIb_β_3_), CD42b (GPIbα) and GPVI was similar in the knockout mice and PF4-Cre^−^ (Wild-type, WT) littermate controls (Fig. 1c). Furthermore, mTOR^−/−^ mice survived at about 100% of the expected Mendelian frequency (Additional file 1: Table S1). These results verify the successful deletion of mTOR in mTOR^−/−^ mice platelets and also suggest that survival was not significantly affected by megakaryocyte- and platelet-specific mTOR deletion.

To determine whether megakaryocyte- and platelet-specific mTOR deletion affects the distribution of cells, including platelets, in peripheral blood, blood from littermate WT and mTOR^−/−^ mice was collected. As shown in Table 1, the platelet counts (PLTs), mean platelet volume (MPV), platelet distribution width (PDW), red blood cell count (RBCs), hemoglobin (HGB) and white blood cell count (WBCs) were normal in mTOR^−/−^ mice (P > 0.05; n = 12).

**Table 1:** Hematologic parameters in wild-type (WT) and mTOR^−/−^ mice

Hematologic parameter	WT (n = 12)	mTOR^−/−^ (n = 12)	P-value
PLTs (K/μL)	856.67 ± 46.78	922.50 ± 33.60	0.265
MPV, fl	5.16 ± 0.058	5.23 ± 0.04	0.360
PDW, fl	5.97 ± 0.08	6.10 ± 0.08	0.219
RBCs (M/μL)	10.34 ± 0.34	10.26 ± 0.25	0.844
HGB (g/dL)	14.67 ± 0.54	14.42 ± 0.42	0.718
WBCs (K/μL)	6.03 ± 0.65	5.04 ± 0.53	0.254

Whole blood from WT or mTOR^−/−^ mice was collected and used for analyzing the hematologic parameters

*PLTs* platelets, *MPV* mean platelet volume, *PDW* platelet distribution width, *RBCs* red blood cells, *HGB* hemoglobin, *WBCs* white blood cells

The data are presented as means ± SEM (P > 0.05 is nonsignificant for all hematologic parameters)

To examine the activity of mTOR^−/−^ platelets under flow conditions, we used a whole-blood microfluidic perfusion system. Platelets from mice blood were pre-incubated and labeled using mepacrine and then perfused on 20 μg/mL or 50 μg/mL type I fibrillar collagen-coated surfaces. The accumulated platelet adhesion area and fluorescence intensity were quantified and as a measure of thrombus formation. For 20 μg/mL type I fibrillar collagen-coated surfaces, smaller thrombi were formed after perfusion with blood from mTOR^−/−^ mice than that from WT mice (Fig. 1d–f). However, for higher concentration (50 μg/mL) collagen-coated surfaces, the defective thrombus formation in mTOR^−/−^ blood was overcame (Fig. 1g–i).

These results suggest that, although the platelets in mTOR^−/−^ mice were produced at normal levels, they have the impaired ability to form thrombi on low-concentration collagen-coated surfaces, which was recovered on higher-concentration collagen-coated surfaces. To verify these results, we perfused reconstituted blood containing the same concentration of calcein-labeled platelets (2.0 × 10^8^/mL) onto 20 μg/mL or 50 μg/mL type I fibrillar collagen-coated surfaces. Similar results were obtained as that in the whole blood experiments (Additional file 1: Figure S1).

FeCl_3_-induced mesenteric arteriole thrombosis models were used to investigate in vivo thrombus formation*.* The ratio of occluded mice showed a significant difference (4/8 for WT vs 2/7 for mTOR^−/−^) for mice with a lower extent of injury, especially within the first 40 min (3/8 for WT vs 0/7 for mTOR^−/−^). Moreover*,* the occlusion time of the injured mesentery arteriole was proportionally longer for mTOR^*−/−*^ mice compared to WT mice with a minor injury. Additionally, there was no significant difference between mTOR^−/−^ mice and WT mice with a higher extent of injury.

In addition, WT and mTOR^−/−^ mice showed dose-dependent responses to the injury. The difference in mTOR^−/−^ mice was far greater than that in WT mice in response to the different extent of injury. The dose effect on the extent of injury in WT mice was observed as follows: the ratio of occluded mice (4/8 for lower extent injury vs 8/12 for higher extent injury, and 3/8 vs 8/12 within the first 40 min) and the occlusion time of the mesentery arteriole response to the varying extent of injury were different (P < 0.05). For the mTOR^*−/−*^ mice: the ratio of occluded mice (2/7 for lower extent injury vs 9/11 for higher extent injury, and 0/7 vs 9/11 within the first 40 min) and the occlusion time of the mesentery arteriole after being subjected to varying levels of injury were remarkably different (P < 0.01) (Additional file 1: Figure S2). These results suggest that in vivo thrombus formation was proportional to the extent of injury in WT and mTOR^*−/−*^ mice, and the dose-dependent effect was far greater in mTOR^*−/−*^ mice than in WT mice in regard to the results of the ratio of occluded mice and the occlusion time of the mesentery arteriole.

Collectively, these results suggest that mTOR positively regulates thrombus formation, both in vitro and in vivo, when perfused on low-concentration collagen-coated surfaces or after being subjected to less severe FeCl_3_-induced injury, respectively. Moreover, mTOR performs these functions in a dose-dependent manner.

### mTOR^−/−^ platelets exhibit impaired aggregation and dense granule secretion (ATP release) after stimulation with low doses of the GPVI agonist CRP or the PAR4 agonist GYPGKF-NH_2_

**Fig. 2:**
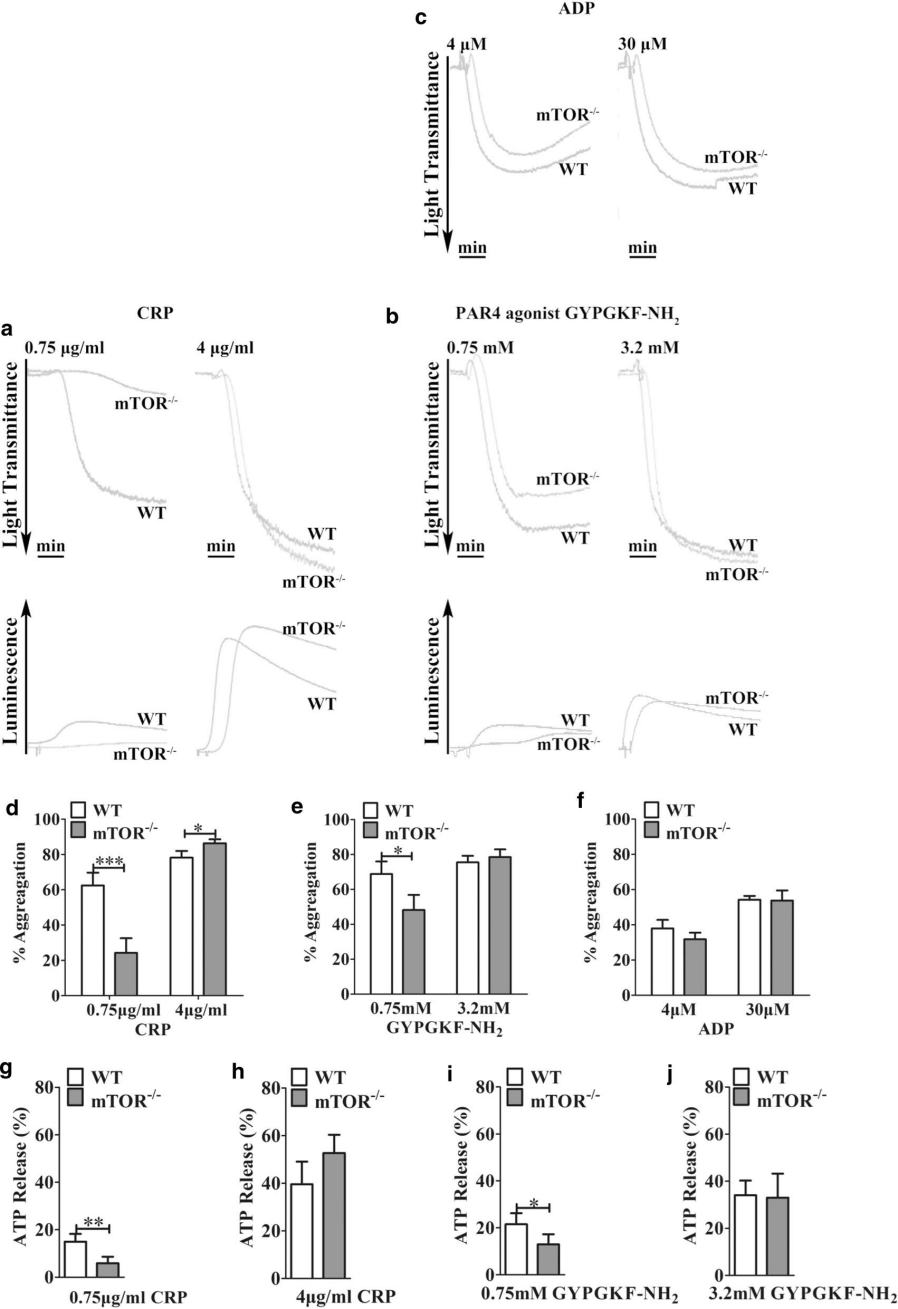
mTOR^*−/−*^ platelets exhibit impaired aggregation and dense granule secretion (ATP release) after stimulation of GPVI with low-dose CRP or stimulation with the PAR agonist GYPGKF-NH_2_. **a** Washed platelets from mTOR^−/−^ mice and WT littermates were challenged with CRP (0.75 μg/mL or 4 μg/mL) or **b** the PAR4 agonist GYPGKF-NH_2_ (0.75 mM or 3.2 mM), and their aggregation and simultaneous ATP release were measured; **c** PRP was stimulated with ADP (4 μM or 30 μM) and platelet aggregation were measured; **d**–**j** The calculated percentages of aggregation and ATP release. Results are presented as means ± SEM from at least three experiments (*P < 0.05, **P < 0.01, paired Student’s *t* test). The arrows show “aggregation” or “ATP Release,” indicating 90% light transmission or 70% ATP release, respectively

To further evaluate the effects of mTOR deletion on platelet activity, we assessed the aggregation and dense granule secretion (ATP release) of platelets after activation with low and high doses of: GPVI agonist CRP (collagen-related peptide) (0.75 μg/mL and 4 μg/mL); PAR4 (protease-activated receptor-4) agonist GYPGKF-NH_2_ (0.75 mM and 3.2 mM); and ADP (4 μM and 30 μM) (Fig. 2a–c). After activation with a low dose of CRP, mTOR^*−/−*^ platelets displayed impaired activity in aggregation (Fig. 2a, d) and dense granule secretion (ATP release) (Fig. 2a, g); however, these deficiencies were overcame by high-dose activation (Fig. 2a, d, h). This pattern was also replicated for GYPGKF-NH_2_, though the effect of mTOR deficiency was less obvious (Fig. 2b, e, i, j). In contrast, the aggregation level of mTOR^*−/−*^ platelets that were induced by ADP (4 μM and 30 μM) was similar to that of WT platelets (Fig. 2c, f). These results suggest that mTOR^*−/−*^ platelets are specifically impaired in their response to activation by GPVI and PAR4 at low concentrations.

### mTOR^−/−^ platelets show impaired activation of α_IIb_β_3_, but normal α-granule secretion after induction with GPVI/PARs agonists at low concentrations

**Fig. 3:**
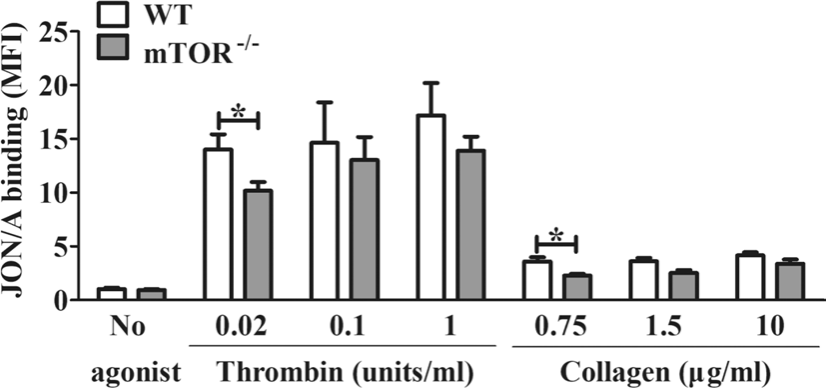
mTOR^*−/−*^ platelets exhibit impaired activation of α_IIb_β_3_ after induction with low concentrations of collagen/CRP or the PAR agonist thrombin. Washed platelets were pre-incubated with phycoerythrin-labeled rat anti–mouse integrin α_IIb_β_3_ mAb (JON/A). Then, they were activated with thrombin, collagen/CRP. The mean fluorescence intensity (MFI) was measured by flow cytometry. Results are expressed as MFI ± SEM (n ≥ 3). The data were analyzed for statistical significance using the Student’s *t* test (*P < 0.05, **P < 0.01)

To further characterize the deficiency in mTOR^*−/−*^ platelets, we used the α_IIb_β_3_-specific antibody JON/A or APC-fibrinogen to assess the activation of integrin α_IIb_β_3_ upon stimulation with increasing doses of the PAR agonist thrombin and the GPVI agonist collagen. mTOR^*−/−*^ platelets showed impaired activation of α_IIb_β_3_ after stimulation with a low doses of thrombin or collagen; however, the levels of activation of α_IIb_β_3_ were not affected by mTOR deficiency, when stimulated at higher concentrations (0.1 and 1 U/mL thrombin; 1.5 or 10 μg/mL collagen) (Fig. 3). However, the expression of P-selectin was not influenced by mTOR deficiency (Additional file 1: Figure S3), suggesting that the effect of mTOR deficiency may be specific for integrin α_IIb_β_3._

The spreading of mTOR^−/−^ platelets on 50 μg/mL [Additional file 1: Figure S4, as well as 10 μg/mL (data not shown)] fibrinogen were enhanced than WT platelets. However, the clot retraction was delayed in mTOR^−/−^ platelets compared with the WT platelets (Additional file 1: Figure S5). Taken together, these results suggested that mTOR may play different roles in early and late α_IIb_β_3_-mediated outside-in signaling (see Additional file 1: Results and Discussion).

### Identification of mTOR-dependent signaling molecules that are modulated in response to low-dose CRP

**Fig. 4:**
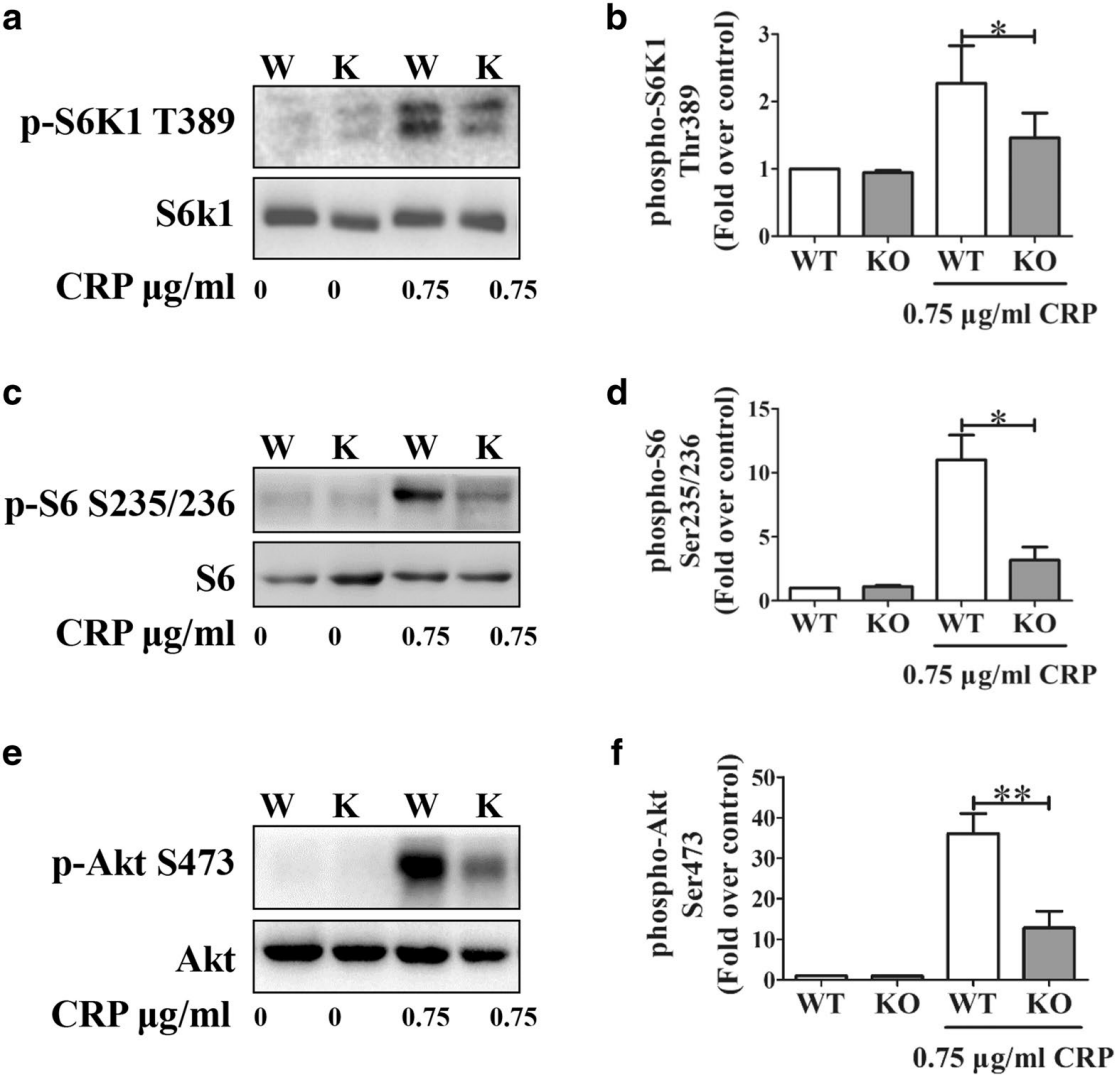
mTOR deficiency impairs low-dose CRP induced phosphorylation of S6K1, S6, and Akt Ser473. Platelets from WT (W) or mTOR^−/−^ (KO, K) mice were stimulated with CRP at the indicated low concentration under aggregating conditions for 5.5 min. Lysates of platelets were immunoblotted with antibodies to **a**, **b** phospho-S6K1 Thr389 and S6K1, **c**, **d** phospho-S6 Ser235/236 and S6, or **e**, **f** phospho-Akt Ser473 and Akt; these images (separated by horizontal white space) were cropped from the different/same gels and full-length/original blots were shown in Additional file 3: Part III. Phosphoprotein levels were normalized to S6K1 levels for panel b, to S6 levels for panel c, and to Akt levels for panels e. Relative values were standardized to 1 in unstimulated WT samples and represent means ± SEM from at least three independent experiments (*P < 0.05, **P < 0.01; paired Student’s *t* test)

To provide a molecular basis for reduced GPVI-mediated mTOR^−/−^ platelet activation, we firstly investigated the phosphorylation of S6K1, S6, and Akt Ser473, each of which has been reported to be regulated by mTORC1 and mTORC2 [4, 15, 16]. The phosphorylation of S6K1 Thr389, S6 Ser235/236 (Fig. 4a–d), and Akt Ser473 (Fig. 4e–f) was significantly decreased in mTOR-deficient platelets in response to low-dose CRP (0.75 μg/mL), which verifies the impaired downstream signaling in mTOR^−/−^ platelets after GPVI stimulation. Our study shows that the phosphorylation of the S6 and Akt Ser473 in mTOR^−/−^ platelets was decreased to approximately 30% of that of WT platelets in GPVI signaling. Moreover, the phosphorylation of S6 and Akt Ser473 was ablated in some samples of mTOR^−/−^ platelets after GPVI stimulation.

Furthermore, apyrase (1 U/mL) decreased the phosphorylation of S6 and Akt Ser473 in mTOR^*−/−*^ platelets after induction with low-concentration collagen (0.8 μg/mL). The phosphorylation level of S6/Akt Ser473 in mTOR^*−/−*^ platelets decreased to a level similar to that of resting mTOR^−/−^ platelets when pre-incubated with apyrase. These findings occurred independently of whether there was substantive S6/Akt Ser473 phosphorylation in mTOR^−/−^ platelets when pre-incubated without apyrase. Conversely, apyrase played a less dramatic role on the phosphorylation of S6 in WT platelets than in mTOR^−/−^ platelets (for unknown reasons), although apyrase decreased the phosphorylation level of Akt Ser473 in WT platelets. The phosphorylation level of S6/Akt Ser473 in apyrase + collagen WT platelets was higher than that in resting WT platelets (Additional file 1: Figure S6). A similar decrease in the phosphorylation of S6 Ser235/236 and Akt Ser473 was also observed in mTOR-deficient platelets in response to low-dose PAR4-agonist GYPGKF-NH_2_ (0.75 mM) (data not shown). These results confirm mTOR deficiency impaired the downstream signaling in platelets after GPVI stimulation.

We also examined effects on the phosphorylation of Lyn, which is activated by GPVI-mediated signals [57]. Lyn phosphorylation was similar in mTOR^−/−^ and WT platelets after induction with low-dose CRP (data not shown) which verifies the specificity of the GPVI-mediated mTOR-dependent signal.

mTOR also interacts with mitogen-activated protein kinases, including Erk, in various cell types [17, 18]. Consistently, the phosphorylation of Erk Thr202/Tyr204 was significantly decreased in mTOR-deficient platelets in response to low-dose CRP or PAR4 agonist (data not shown). Moreover, a similar decrease in the phosphorylation of these downstream signaling molecules in response to low-dose collagen (0.8 μg/mL) was observed in WT platelets after treatment with the mTOR inhibitor Torin1, although almost no change was observed for mTOR^−/−^ platelets after the same treatment (Additional file 1: Figure S7). These results verify the impaired downstream signaling molecules in mTOR^−/−^ platelets after low-dose GPVI stimulation.

**Fig. 5:**
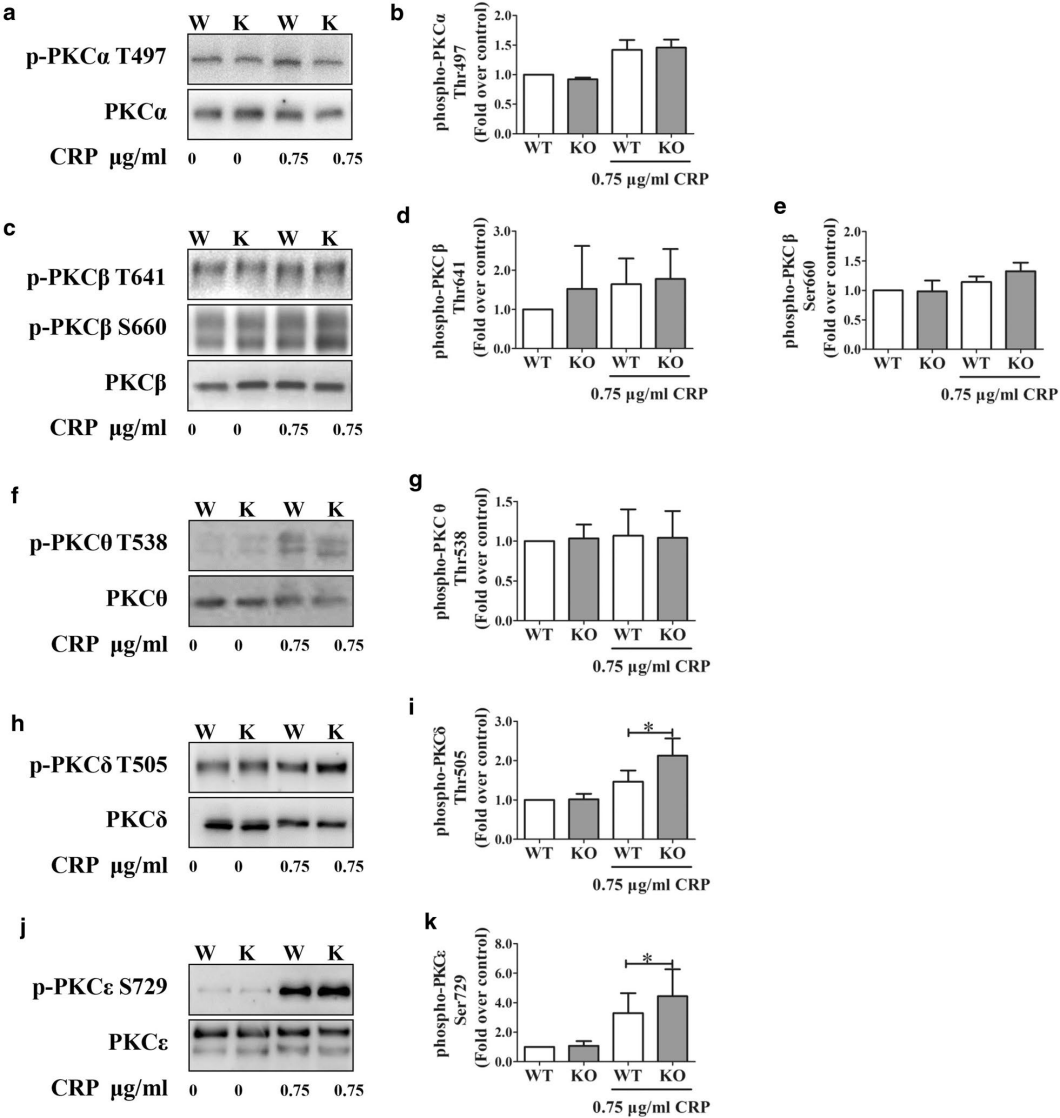
mTOR regulates CRP-induced phosphorylation of PKCs. Platelets from WT (W) or mTOR^−/−^ (KO, K) mice were stimulated with CRP at the indicated low concentration for 5.5 min. Lysates of platelets were immunoblotted with antibodies against **a**, **b** phospho-PKCα Thr497 and PKCα, **c**, **d** phospho-PKCβ Thr641 and PKCβ, **c**, **e** phospho-PKCβ Ser660 and PKC β, **f**, **g** phospho-PKCθ Thr538 and PKCθ, **h**, **i** phospho-PKCδ Thr505 and PKCδ, and **j**, **k** phospho-PKCε Ser729 and PKCε; these images (separated by horizontal white space) were cropped from the different/same gels and full-length/original blots were shown in Additional file 3: Part III. Phosphoprotein levels were normalized to total protein levels and were standardized to 1 in unstimulated WT samples. Values represent means ± SEM from at least three independent experiments (*P < 0.05, **P < 0.01; paired Student’s *t* test)

PKCs also have been reported to be regulated by mTOR or mTORC2 in other cell types [4] and to mediate platelet aggregation, dense- and alpha- granule secretion, α_IIb_β_3_ activation, and spreading on fibrinogen [9, 10, 13, 19–34]. Furthermore, the expression of cPKCα, cPKCβ, nPKCδ and nPKCθ have been observed in human platelets in many studies [9, 10, 13, 19–34]. The phosphorylation of Thr497 [27] in the activation loop of PKCα, Thr505 [35] in the activation loop of PKCδ, and Thr538 [40, 41] in the activation loop of PKCθ, is thought to be critical for kinase activity [42]. cPKC and nPKC must be phosphorylated at both their turn motif and hydrophobic motif to achieve catalytic competence. The phosphorylation of PKCβ Thr641, PKCβ Ser660 [27], and PKCε Ser729 [20] is also essential for activation [42]. Thus, to comprehensively examine the role of PKCs in platelet activation by low-dose CRP, we assessed the phosphorylation of an array of PKCs in WT and mTOR-deficient platelets. The phosphorylation of PKCα Thr497 (Fig. 5a, b), PKCβ Thr641 and Ser660 (Fig. 5c, e) and PKCθ Thr538 (Fig. 5f, g) were unaffected in low-dose CRP-activated mTOR^−/−^ platelets. Moreover, the cPKC activity, which was detected by a phosphorylated cPKC substrate antibody, showed little change in mTOR-deficient platelets when induced by a low concentration of collagen (data not shown).

However, the phosphorylation of PKCδ Thr505 (Fig. 5h, i) and PKCε Ser729 (Fig. 5j, k) was enhanced by low-dose CRP in mTOR-deficient platelets. Torin1 increased the phosphorylation of PKCδ Thr505 and PKCε Ser729 in WT platelets after low-dose collagen stimulation with GPVI (Additional file 1: Figure S7). Moreover, the increased phosphorylation of PKCδ Thr505 and PKCε Ser729 in mTOR^−/−^ platelets after stimulation with low-dose collagen was restored by the addition of Torin1, which also supports the possibility that PKCδ and PKCɛ are regulated by mTOR (Additional file 1: Figure S7). Furthermore, ADP restored the decreased phosphorylation of S6 Ser235/236, Akt Ser473, as well as increased phosphorylation of PKCδ Thr505 and PKCε Ser729 in mTOR^−/−^ platelets when stimulated with GPVI agonist collagen at a low dose (Additional file 1: Figure S7).

**Fig. 6:**
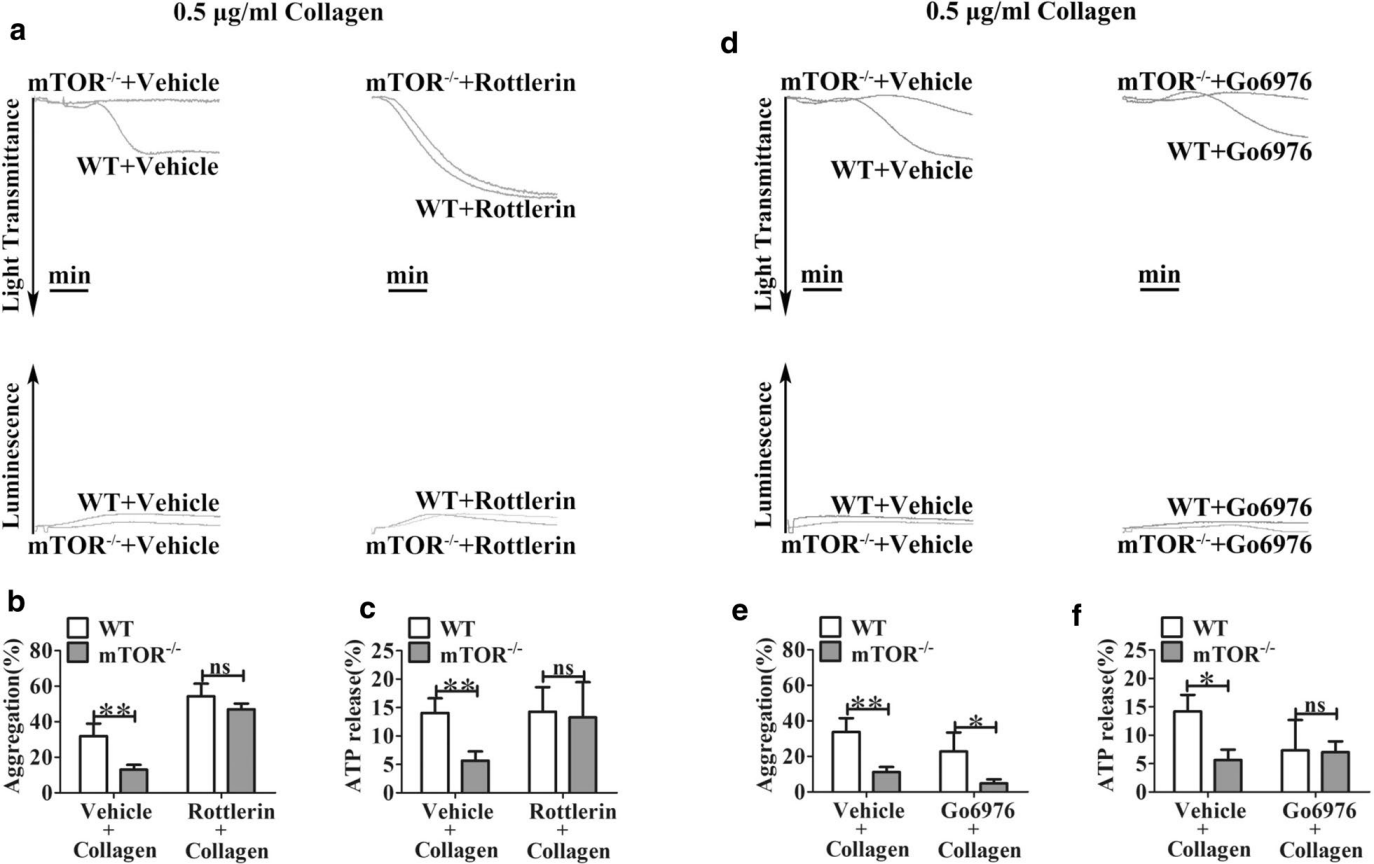
The PKCδ inhibitor rottlerin restores the aggregation and dense granule secretion (ATP release) of mTOR^*−/−*^ platelets in response to low-dose collagen. **a**–**c** Wash platelets were pre-incubated with DMSO (vehicle), rottlerin (5 μM) or **d**–**f** Go6976 (40 nM) for 10 min and then stimulated with collagen at the indicated low dose (0.5 μg/mL). The PKCδ inhibitor rottlerin, but not the PKCα/β inhibitor Go6976, restored the full aggregation of mTOR^*−/−*^ platelets in response to low-dose collagen (0.5 μg/mL). Data from at least three independent experiments were quantified and expressed as means ± SEM (*P < 0.05, **P < 0.01, Paired Student’s *t* test). The arrows show “aggregation” or “ATP Release,” indicating 90% light transmission or 70% ATP release, respectively

To further examine the potential role of PKCs on mTOR-regulated platelet activation, we assessed the effects of treating platelets with the PKCδ inhibitor rottlerin and the PKCα/PKCβ1 inhibitor Go 6976. Rottlerin potentiated the aggregation of WT platelets and restored the full aggregation and dense granule secretion (ATP release) of mTOR^*−/−*^ platelets in response to low-dose collagen (0.5 μg/mL) (Fig. 6a–c), while Go 6976 did not restore them (Fig. 6d–f). However, Go 6976 exhibited a trend of decrease aggregation and dense granule secretion (ATP release) (Fig. 6d–f).

PKC isoform-specific peptide inhibitors, such as δV1-1, ɛV1–2, θV1-1, αC2–4, βC2–1, and βC2–4, are reported to inhibit the interaction of PKCs with their specific RACK adaptor proteins, which essentially block them from translocating to their specific target substrates [21, 41, 44–48, 58–60]. Moreover, peptides δV1-1 [21] and θV1-1 [41] were used in studying the role of PKC isoforms in platelets. The use of peptide ɛV1–2 [20] also has been mentioned in a study of platelets, although the data were not shown. The PKCδ peptide inhibitor δV1-1 rescued the aggregation of mTOR^*−/−*^ platelets in response to low-dose collagen, whereas the PKCε peptide inhibitor had a similar but minimal role in rescuing the aggregation of mTOR^−/−^ platelets in response to low-dose collagen (Additional file 1: Figure S8).

These results suggest that PKCδ/ε, especially PKCδ, may be involved in low-dose GPVI agonist-induced mTOR-dependent signaling but PKCα/β and PKCθ are not involved.

## Discussion

Whether the inhibitor of mTORC1-Sirolimus (rapamycin) enhances or impairs thrombus formation is a subject of debate [13, 61, 62]. Many studies using rapamycin suggest that mTOR plays a positive role in thrombopoiesis at different stages of thrombocytopoiesis in vivo or in vitro [61–64]. However, rapamycin has also been reported as an option for the therapy of idiopathic thrombocytopenic purpura in clinical trials [13]. Since genetic deletion of mTOR in mice affects the animal’s survival [14], it is difficult to resolve these discrepancies using standard knockout methods. Therefore, we established megakaryocyte/platelet-specific mTOR deletion mice. Our results confirm the role of mTOR in low-dose-induced platelet aggregation and in in vitro thrombus formation (Additional file 1: Figure S9, as well as see Additional file 2).

Whole-blood/reconstituted-blood from mTOR^−/−^ mice displayed deficiencies in thrombus formation when perfused in lower concentration (20 μg/mL) collagen-coated flow chambers. These deficiencies in mTOR^−/−^ bloods were overcame when perfused in higher concentration (50 μg/mL) collagen-coated flow chambers.

These results demonstrate that mTOR functions as a positive regulator of thrombus formation when perfused in vitro on low-concentration collagen-coated surfaces, and that mTOR plays this role in a collagen dose-dependent manner.

Similar to the results found in thrombus formation in vitro, mTOR positively regulates thrombogenesis in vivo after being subjected to less severe FeCl_3_-induced injury, and mTOR performs this function in a dose-dependent manner, which is dependent on the extent of FeCl_3_-induced injury. Platelet-specific deletion of the Raptor gene, a component of mTORC1, has also been shown to impair thrombus formation in vivo [65]. The deletion of mTOR would be assumed to affect both mTORC1 and mTORC2 signaling. Nonetheless, our results could be explained by effects on one or both complexes. Our results also showed that in vivo thrombus formation in WT and mTOR^*−/−*^ mice were proportional to the extent of injury, and the dose-dependent effect was far greater in mTOR^*−/−*^ mice than in WT mice. This dose-dependent effect in the FeCl_3_-induced mesenteric arteriole thrombosis models has been reported previously [66–68]. However, tail bleeding times did not differ significantly between the mTOR^−/−^ mice and WT mice (data not shown); the mechanism of this discrepancy is currently unknown, though the outcomes of tail injury models are known to be variable [69]. Therefore, much work needs to be done to increase our understanding of the detailed mechanisms of thrombus formation in vivo and in vitro and hemostasis.

Our results demonstrate that mTOR^−/−^ platelets show impaired thrombus formation when perfused on low-concentration collagen-coated surfaces, although mTOR^*−/−*^ mice display normal hematopoietic parameters. The platelet counts were normal in the mTOR^−/−^ mice, which is similar to findings in Raptor^−/−^ mice [65], though the results differ in that mTOR deletion had no observable effect on the mean platelet volume (MPV), whereas Raptor^−/−^ mice displayed a reduced MPV. The differences between these results may be explained by a combination of different mechanisms for the inhibition of mTOR in vivo, including a reduction in the translation activity and an increase in the lifespan [4, 70–74], or to the compensatory activation of PI3K signaling [75]. Other groups have also reported that mTOR^l^°^xp/l^°^xp^Mx1-Cre^+^ (mTOR^−/−^) [76] or Rosa26-CreERT2^+^, TSC1^l^°^xp/l^°^xp^ (TSC1^−/−^) [77] mice are deficient in platelet production. The discrepancies may be explained by the differences in the conditional knockout models: normal platelet counts were observed for Raptor^fl/fl^CreER ^+TAM^ (Raptor-deficient) mice [71] and Raptor^fl/fl^ PF4-Cre + (Raptor^−/−^) [65], while other groups report that Raptor^l^°^xp/l^°^xp^Mx1-Cre^+^ (Raptor^−/−^) mice show deficient platelet production [78]. PF4-Cre mice were used to generate megakaryocyte/platelet-specific knockout mice [51], whereas Mx1-Cre was induced by polyinosinic-polycytidylic acid [79] and used to generate knockout cells during development, including hematopoietic stem cells [78]. Further work on the complex mechanisms of mTORC1 and mTORC2 may help to elucidate the role of mTOR at different stage of thrombopoiesis.

The dose-dependent deficiency in mTOR^−/−^ platelets during thrombus formation in vitro, particularly when grown on low-concentration collagen-coated surfaces, may be explained by platelet function (Additional file 1: Figure S9, as well as see Additional file 2).

Our results also demonstrate that isolated mTOR^−/−^ platelets display impaired thrombus formation after blood perfusion on micro-flow chambers which were coated with low-concentration fibrillar collagen. Consistently, GPVI- and PAR4-mediated aggregation was impaired in mTOR^−/−^ platelets upon activation with low concentrations of the agonists CRP and GYPGKF-NH_2_, while the aggregation level of mTOR^*−/−*^ platelets that were induced by ADP was approximately consistent to that of WT platelets. No deficiency in aggregation was detected in mTOR^−/−^ platelets when higher doses of these agents were tested, which suggests that the phenotype of mTOR^−/−^ platelets is dose-dependent. Similar to our findings, Musumeci and colleagues showed a dose-dependent difference in DUSP3-deficient platelets: the platelet aggregation was impaired for low-dose CRP-induced DUSP3-deficient platelets, but no deficiency in aggregation was detected for higher dose CRP-induced DUSP3-deficient platelets [80]. Moreover, the levels of α_IIb_β_3_ activation were decreased in mTOR^−/−^ platelets compared to WT platelets upon stimulation with low-dose GPVI-dependent collagen or PAR-dependent thrombin. However, there was almost no difference between the mTOR^−/−^ and WT platelets in the activation of α_IIb_β_3_ after stimulation with higher concentrations. Additionally, mTOR^−/−^ platelets displayed increased spreading while reducing clot retraction; however, this discrepancy in the literature may be explained by different molecular mechanisms between these processes [81–83] (see Additional file 1: Results and Discussion). However, future work is needed to pinpoint mTOR-regulated molecules in outside-in signaling. Our data also revealed that mTOR plays a negative role in regulating platelet spreading on collagen-coated surfaces, which is similar to the results of spreading on fibrinogen. These results suggest that mTOR may plays different roles in early and late outside-in signaling.

Platelets contain several types of secretory granules, most notably α-granules, dense granules, and lysosomes. α-granules contain P-selectin, fibrinogen, and other proteinaceous components; lysosomes contain proteolytic enzymes; and dense granules contain ATP, ADP, calcium, serotonin, and other molecules. Our results suggest that the regulation of dense granule secretion (ATP release) is an essential role of mTOR in platelets that are stimulated by GPVI agonists. This conclusion is based upon the following observations: (1) The expression of P-selectin was almost not influenced by mTOR deficiency in platelets after induction by agonists; (2) the defect in the aggregation of mTOR^−/−^ platelets induced by lower dose GPVI-dependent collagen was rescued by supplementation with ADP, while the aggregation of WT platelets induced by low-dose GPVI-dependent collagen was decreased to a level similar to that of mTOR-deficient platelets when apyrase was applied (data not shown); and (3) ADP restored the decreased phosphorylation of S6 Ser235/236, Akt Ser473, as well as the increased phosphorylation of PKCδ Thr505 and PKCε Ser729 in mTOR^−/−^ platelets, although it is unclear why the phosphorylation levels of these molecules were not significantly increased in WT platelets after exogenous ADP was added (Additional file 1: Figure S7).

Our results demonstrate that the phosphorylation of Erk Thr202/Tyr204 was significantly decreased in mTOR-deficient platelets or in WT platelets after treatment with the mTOR inhibitor, Torin1, when stimulated with GPVI/PARs agonist (data not shown). There is synergistic crosstalk between mTOR and MAPKs in other cell types [84]. For example, some studies observed MAPKs regulated mTOR (mTORC1) [63, 85, 86], while other studies found that mTOR (mTORC2) regulated MAPKs [87–89]. Interestingly, PKCs have been also been reported to be regulated by mTOR or mTORC2 in other cell types [4], and it is documented that MAPKs play important roles in regulation by PKCs in platelet activation [2]. Based on the literature mentioned above as well as our results, we hypothesize that mTOR or mTORC2 regulates Erk (possibly through PKCs).

Analysis of signaling molecules demonstrated that mTOR deficiency impaired the phosphorylation of S6 Ser235/236 and Akt Ser473 in platelets after low-dose CRP/collagen stimulation with GPVI. The phosphorylation of the S6 Ser235/236 and Akt Ser473 in mTOR^−/−^ platelets was decreased to around 30% of that of WT platelets, and the phosphorylation of these substrates was ablated in some samples of mTOR^−/−^ platelets pre-incubated without apyrase.

Moreover, a similar decrease in the phosphorylation of these downstream signaling molecules in response to low-dose collagen (0.8 μg/mL) was observed in WT platelets after treatment with the mTOR inhibitor Torin1, while almost no change was observed for mTOR^−/−^ platelets after the same treatment. As observed from the sample bands, Torin1 may have exerted an effect on mTOR^−/−^ platelets regarding the phosphorylation of these downstream signaling molecules; however, there was no significant difference between mTOR^−/−^ platelets pre-incubated with Torin1 and mTOR^−/−^ platelets pre-incubated without Torin1. Torin1 ablated the phosphorylation of S6/Akt Ser473 in WT platelets, which is similar to findings provided by Moore and colleagues [9]. A reason for these phenomena is that as a chemical inhibitor, Torin1 also plays a nonspecific role on other kinases. For example, Torin1 exhibits a weaker influence on PI3K/Akt [90]. We further confirmed the mTOR deficiency ablated phosphorylation of the substrates when apyrase was used to hydrolyze the released ADP. These findings occurred independently of substantive S6/Akt Ser473 phosphorylation in mTOR^−/−^ platelets in the absence of exogenous apyrase. Additionally, exogenous ADP restored the decreased phosphorylation of S6 Ser235/236 and Akt Ser473 in mTOR^−/−^ platelets when stimulated with low-concentration GPVI agonist collagen. In addition, mTOR exerted almost no effect on ADP-induced platelet activation. Please see Additional file 1: Figure S7, S6 and Fig. 2c in order.

These results suggest that the released ADP activated P_2_Ys, can bypass mTOR complexes and phosphorylate Akt Ser473 or S6 Ser235/236 (possibly through ERK [63, 72, 73, 75, 85, 86]) in mTOR^−/−^ platelets when induced by low concentrations of GPVI-agonist/PAR4-agonist (PAR4 agonist GYPGKF-NH_2_ data not shown); these phosphorylation events can be amplified by an ADP secretion cascade. This may be the primary, or even the sole reason, why phosphorylation of the substrates is not typically ablated in mTOR^−/−^ platelets when pre-incubated without apyrase. Interestingly, previous reports using blockers of ADP-receptor or apyrase have implicated that the direct activation of S6K1/S6 [10, 13, 91] and Akt Ser473 (as well as Erk, [92, 93]) by GPVI or PARs signaling mainly exists in normal platelets when stimulated with GPVI/PARs. In addition, it is well known that the ADP secretion induced by agonists and the subsequent activation of P_2_Ys would amplify phosphorylation signaling (including Akt Ser473, Erk) [2, 92, 94]. However, whether other molecules beyond ADP secretion-P2Ys are involved is unknown. These interesting phenomena as well as their mechanisms should be studied in future work.

These results also are consistent with the deficiency in mTOR signaling.

We further assessed the effect of mTOR deficiency on the phosphorylation of several PKC isoforms, which are classified by the structure of their regulatory domains. The expression of the cPKC (classical PKC) isoforms cPKCα and cPKCβ, and the nPKC (novel PKC) isoforms nPKCδ and nPKCθ have been observed in human platelets in many studies [33, 35, 36]. Additionally PKCε is expressed in human megakaryocytes but not in human platelets [28, 36, 38], though mouse platelets also express PKCε [28, 39]. We therefore assessed the effects of CRP on each of these platelet-expressed PKCs. Our results demonstrate that the phosphorylation of both PKCδ and PKCε was enhanced after low-dose CRP stimulation. PKC enzymes have been proposed to regulate steps in the process of platelet activation, such as the mobilization of calcium ion, α_IIb_β_3_-mediated signaling; exocytosis or the secretion of granules; filopodia formation; and adhesion to the extracellular matrix [20, 23, 25, 29, 33, 35, 36, 95]. Additionally, it has been suggested that cPKCs positively regulate platelet activation and thrombus formation, while the nPKC isoforms have been proposed to execute a negative regulatory role on platelet activation, at least in some cases [24, 96].

To verify the role of PKCδ in mTOR-dependent collagen response, we assessed the effects of the PKCδ inhibitor rottlerin. Our results demonstrate that rottlerin restored full aggregation and dense granule secretion (ATP release) of mTOR-deficient platelets in response to low-dose collagen. The selective PKCδ peptide inhibitor also restored the aggregation of mTOR^−/−^ platelets in response to low-dose collagen, and exerted smaller but similar role on the dense granule secretion of mTOR^*−/−*^ platelets. We also found that εV1-2 minimally rescued the aggregation of mTOR-deficient platelets in response to low-dose collagen. Given that PKCε has widely been reported to be a negative regulator of ADP/P2Ys-mediated platelet activation [20, 97], these results are consistent with current understanding of εV1-2′s.

These results suggest that PKCδ/ɛ, especially PKCδ but not PKCα/β and PKCθ, may be involved in low-dose GPVI-mediated mTOR-dependent signaling (Additional file 1: Figure S9 as well as see Additional file 2).

## Conclusions

In conclusion, we demonstrated that depending on the collagen concentration or extent of injury, mTOR functions as a positive regulator in thrombus formation in vitro when perfused on low-concentration collagen-coated surfaces or in vivo after being subjected to less severe FeCl_3_-induced injury. mTOR was also found to exert a positive role in low-dose activation of GPVI in platelets in a dose-dependent manner, although the effect of mTOR deficiency was less dramatic, different experimental designs yielded similar results. Further work is required for a more complete understanding of the network of signaling mediators that are involved in this complex process.

## Supplementary Information

**Additional file 1.** Additional Data S I, II

**Additional file 2.** Additional Information Part I

**Additional file 3.** Additional Information Part II & III

## Data Availability

The data used to support the findings of this study are available from the corresponding author upon request.
