# Supplementary material for: mTOR regulates GPVI-mediated platelet activation
Source: J Transl Med. 2021 May 10;19:201. doi: 10.1186/s12967-021-02756-y (PMC8111939; doi:10.1186/s12967-021-02756-y)
Supplement: Supplementary file 1 — Additional file 1. Additional Data S I, II [file 12967_2021_2756_MOESM1_ESM.docx]

**Additional files­­(Additional Data S I, II and Additional Information Part I, II & III)**

**mTOR regulates GPVI-mediated platelet activation**

Longsheng Wang^1^*****, Gang Liu^1, 5^*****, Nannan Wu^1^*****, Baiyun Dai^1^, Shuang Han^1^, Qiaoyun Liu^2^, Fang Huang^2^, Zhihua Chen^3^, Weihong Xu^4^, Dajing Xia^2^, Cunji Gao^1, 6 #^

^1^ Chronic Disease Research Institute, Department of Nutrition and Food Hygiene, Zhejiang University School of Public Health, 866 Yu-Hang-Tang Road, Hangzhou 310058, China.

^2^ Department of Toxicology, Zhejiang University School of Public Health, 866 Yu-Hang-Tang Road, Hangzhou 310058, China.

^3^ Department of Respiratory Medicine, Second Affiliated Hospital, Zhejiang University School of Medicine; 88 Jiefang Road, Hangzhou 310009, China.

^4^ Zhejiang Hospital, 12 Lingyin Road, Hangzhou 310013, China.

^5^ Department of pharmacology, School of Basic Medical Sciences, Guizhou Medical University, Guiyang, Guizhou, China.

^6^ Blood Research Institute, Blood Center of Wisconsin, Milwaukee, Milwaukee, WI 53201, USA.

***Longsheng Wang and Gang Liu and Nannan Wu contributed equally to this work.**

^#^**Address for correspondence:**

Cunji Gao, PhD, Chronic Disease Research Institute, Department of Nutrition and Food Hygiene, Zhejiang University School of Public Health, 866 Yu-Hang-Tang Road, Hangzhou 310058, China, Tel: +86 571 882 085 91, Fax: +86 571 882 085 91, E-mail: zjuc239@163.com.

**Additional file 1: Additional Data S I**

**mTOR regulates GPVI-mediated platelet activation:**

**mTOR appears to play different roles in early and late outside–in signaling**

**Methods**

***Ferric chloride (FeCl_3_) injury thrombosis models***

Arterial arteriole thrombosis models were carried out and developed as previously described [[1](#_ENREF_1), [2](#_ENREF_2)]. Briefly, for the higher extent injury experiments, jugular veins of anesthetized mice were surgically exposed. Then, the veins were catheterized with a PE10 canule (Becton Dicknson). Fluorescently labeled murine platelets (about 1*10^8^ platelets) were injected into the catheter. The mice intestinal mesenteries were surgically exposed, and an arteriole (approximately 60–80 μm in diameter) was selected under the microscope. A 1.0 × 1.0 mm ﬁlter paper saturated with 10% FeCl_3_ was applied to the surface of the arteriole to induce the injury for 3.0 minutes, and blood flow in the injured arteriole was monitored under a Nikon ﬂuorescence microscope (ECLIPSE Ni-U) equipped with a Nikon DS-Qi1-U3. The process of lower extent injury was similar to that of higher extent injury, with some modifications: fluorescently labeled murine platelets (about 1*10^8^ platelets) were gently and immediately transferred to the body via tail vein injection to decrease injuries, and a 0.5 × 1.0 mm ﬁlter paper saturated with 3.5% FeCl_3_ was applied to induce a lower extent of injury on the surface of the arteriole for 10 seconds. Occlusion time was deﬁned as cessation of arteriole blood flow (> 1 min) or a predetermined time (40 or 85.6 min, for higher or lower extent injury, respectively).

***Preparation of red blood cells and measurement of platelet adhesion and aggregation under flow using reconstituted blood***

Red blood cell preparation was carried out as previously described [[3](#_ENREF_3), [4](#_ENREF_4)]. Briefly, the remaining blood was collected during the platelet preparation step after PRP collection and then washed with Tyrode’s buffer containing ACD by centrifugation at 200 × *g* for 20 min at 20°C. The cells were then washed once with Tyrode’s buffer containing ACD by centrifugation at 700 × *g* for 20 min at 20°C. When required, they were mixed with calcein-labeled platelets to form reconstituted blood with a hemocrit of 45%. The washed platelets were resuspended (2 × 10^8^ platelets/mL, final concentration) and 1 mM CaCl_2_ was added. Heparin (7.5 IU/mL) was added into the reconstituted blood during studies in which the concentration of platelets was 2 × 10^8^ platelets/mL. The perfusion step was as described in “In vitro thrombus formation under flow conditions.”

***Platelet spreading***

Platelet spreading was assessed as previously described [[5](#_ENREF_5)]. BSA (25 μg/mL), human fibrinogen (10, 50 μg/mL) or collagen (5, 20, 50 μg/mL) were used to coat glass coverslips overnight. Then, 200 μL of washed platelets (2.0 × 10^7^/mL) were pre-incubated with calcium ions in the absence or presence of thrombin (0.01 U/mL) for 5 min at 37°C, and were added to the surface of coverslips and adhered to BSA, fibrinogen or collagen for the indicated times at 37°C. The adherence time to BSA, or to fibrinogen in the presence of thrombin was 30 minutes, the adherence time to collagen was 45 minutes, and the adherence time to fibrinogen in the absence of agonists (e.g., thrombin) was 60 minutes. The adhered platelets were fixed in 2% paraformaldehyde, and 0.1% Triton X-100 was used to permeabilized the platelets. Then, the adherent platelets were stained with fluorescein-labeled phalloidin. An inverted fluorescence microscope (Nikon Ti-S, Tokyo, Japan) equipt with a Nikon DS-Qi1-U3 camera was used to observe the adherent platelets. The images and the number of platelets adhering on collagen were analyzed using NIS-D software (Nikon).

***Clot retraction***

Clot retraction using mice platelets was assessed as previously described [[6](#_ENREF_6)]. Washed platelets were adjusted to a concentration of 2 × 10^8^/mL, and then 1 mM CaCl_2_ and 400 µg/mL soluble fibrinogen were added. 0.2 U/mL thrombin was used to initiate clot formation, which was incubated at 37°C. The extents of retraction were quantified using Image J software and expressed as: final clot area/initial clot area.

**Statistical analysis**

The results are expressed as means ± SEM. The unpaired or paired Student *t*-tests or Kruskal-Wallis test with Dunn’s multiple-comparison post-test was used to evaluate the statistical signiﬁcance of the data.

**Results**

Integrin α_IIb_β_3_, which is the most abundant platelet surface protein, mediates inside-out and outside-in signaling. Spreading on immobilized ﬁbrinogen and fibrin clot retraction are processes that occur in early and late outside-in signaling, respectively, and are mediated by α_IIb_β_3_ [[7](#_ENREF_7), [8](#_ENREF_8)]. Furthermore, integrin α_2_β_1_ plays a predominant role in platelet spreading on collagen-coated coverslips [[9](#_ENREF_9)]. However, the role of mTOR in α_IIb_β_3_-related signaling and the potential contribution of integrin α_2_β_1_ in mTOR-dependent collagen-mediated spreading are not well known due to the lack of gene knockout technology studies. Therefore, we investigated whether the spreading and clot retraction are altered in mTOR**-**deficient platelets. mTOR^−/−^ platelets displayed increased filopodial extensions on 50 μg/mL (Additional file 1: Figure S4 online, as well as 10 μg/mL (data not shown)) immobilized fibrinogen in the absence of agonists. Additionally, mTOR^−/−^ platelets displayed increased spreading on 20 μg/mL (Additional file 1: Figure S4 online) and 50 μg/mL collagen-coated surfaces, however, the spreading of mTOR^−/−^ platelets on 5 μg/mL collagen-coated was similar to that of WT platelets (50 μg/mL and 5 μg/mL data not shown). Moreover, mTOR deficiency also increased the number of adherent platelets on 20 μg/mL collagen-coated surface (Additional file 1: Figure S4 online, as well as 5μg/mL and 50 μg/mL (data not shown)). In contrast, clot retraction (at 45 min, Additional file 1: Figure S5 online) was delayed in mTOR^−/−^ platelets compared with WT platelets. Taken together, these results suggest that mTOR may play different roles in early and late α_IIb_β_3_-mediated outside-in signaling.

Additional file 1: Figure S4 here

Additional file 1: Figure S5 here

**Discussion**

The above data reveal that mTOR plays an important role in α_IIb_β_3_-mediated outside-in signaling.

mTOR-deficient platelets shown increased spreading on immobilized fibrinogen when compared to the WT platelets. Unlike our results, recently, other group using the AZD8055, an mTOR inhibitor, demonstrate that mTORC2 inhibition does not alter PI3Kβ-dependent platelet spreading on fibrinogen[[10](#_ENREF_10)], however, mTOR knockout and inhibitor effects are likely to be different.

mTOR^−/−^ platelets displayed increased spreading while reducing the clot retraction. The discrepancy in the literature may be explained by different molecular mechanisms between spreading and clot retraction. Du’s group illustrated a calpain-dependent switch mechanism that controls spreading and clot retraction [[7](#_ENREF_7)]. Briefly, activation of a_IIb_β_3_ induces Rac- and Cdc 42-dependent spreading signals, calpain activation and phosphorylation of the cytoplasmic domain of β_3_ at Y579, which protects the C-terminal domain of β_3_ from calpain cleavage and results in RhoA-dependent cell retraction inhibition. After cell spreading, dephosphorylation of β_3_ Y759 occurs and then β_3_ cleavage is mediated by calpain, which result in relief of the inhibition of clot retraction. RhoA-dependent clot retraction is then activated, and thus the outside-signaling is switched from spreading to retraction [[7](#_ENREF_7)]. The same group also observed that the interaction of Ga13-integrin affects this switch process [[8](#_ENREF_8), [11](#_ENREF_11)]. Interestingly, mTORC2 also regulates Rho GTPases (e.g., RhoA, Rac, and Cdc42) [[12](#_ENREF_12)]. Therefore, future work is needed to pinpoint the mTOR-regulated molecules in outside-in signaling. Our data also revealed that mTOR plays a negative role by regulating platelet spreading on collagen-coated surface, which is similar to the effects of spreading on fibrinogen.

**Additional file 1: Additional Data S II**

**Table S1.** Mendelian breeding ratios of mTOR^−/−^ mice.

| **Genotype** | | **mTOR^fl/fl^**  **and**  **mTOR^fl/wt^** | **Cre^+/−^**  **mTOR^fl/wt^** | **Cre^+/−^**  **mTOR^fl/fl^** | **Total** |
| --- | --- | --- | --- | --- | --- |
| Expected Frequency | | 50% | 25% | 25% | 100% |
| Actual | Count | 219 | 114 | 111 | 444 |
|  | Frequency | 49.32% | 25.68% | 25.00% | 100% |

**Additional Data S II: Figure S1-9**

**

**

**Figure S1.**

**Representative photomicrographs of the progression of adhesion and aggregation of WT and mTOR^−/−^ platelets (in reconstituted blood) on collagen-coated surfaces.** Washed platelets (2 × 10^8^ platelets /mL) were labeled with calcein and then mixed with red blood cells (hematocrit 45%) supplemented with 1 mM CaCl_2_ and heparin (7.5 IU/mL). (a–c) Reconstituted blood was perfused over a 20 μg/mL or (d–f) 50 μg/mL type I fibrillar collagen-coated surface, and the fluorescent platelets adhering to coated Bioflux micro flow chambers were recorded by video. Bioflux software (Fluxion) was used to quantify the coverage surface area and total integrated fluorescence, as a measure of platelet thrombus formation. Results are expressed as the mean percentage of surface coverage (left panels) or mean integrated fluorescence intensity (right panels) ± SEM (n ≥ 3 per group; * indicates P < 0.05, Paired Student’s *t* test).


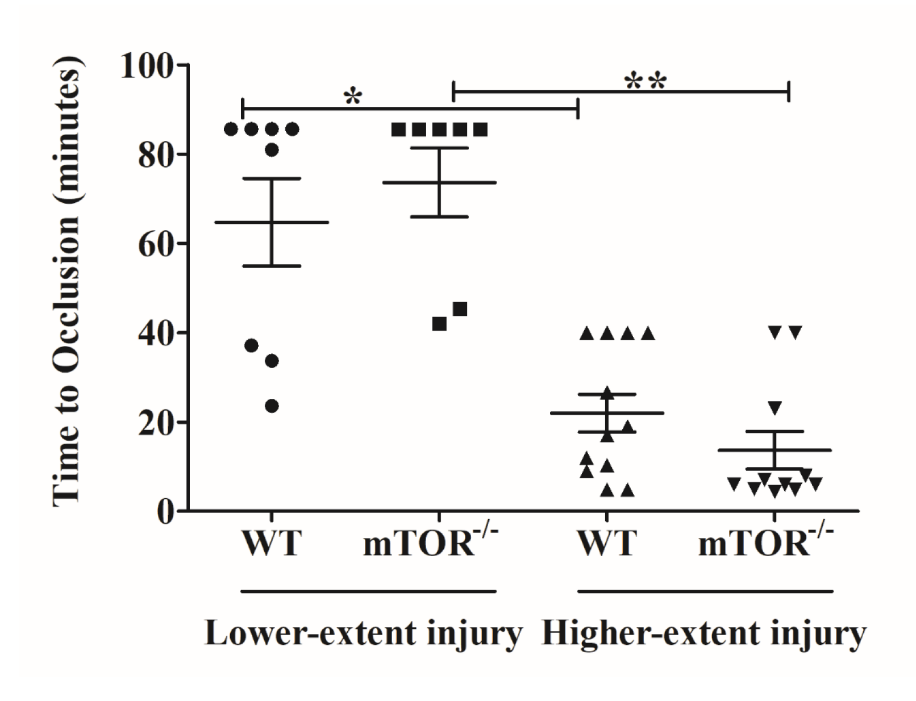


**Figure S2.** **Assessment of the role of mTOR in thrombus formation *in vivo*.**

(a) Mesenteric arteries from WT or mTOR^−/−^ mice were subjected to a lower or (b) higher extent of injury, and then occlusion times were observed. Dot-plots show the occlusion times for arterioles. The median occlusion time is indicated by the horizontal lines (the nonparametric Kruskal-Wallis test with Dunn’s multiple-comparison post-test was used to determine the statistical significance). Occluded mice were determined as the mice that blood flow in arterioles stopped (> 1 min) within a predetermined time and the ratios of occluded mice were calculated simultaneously. The number of occluded mice can also be observed from the dot-plots. (a) The median occlusion time for WT mice (n = 8) was 64.81 ± 9.83 min, while that of mTOR^−/−^ mice (n = 7) was 73.76 ± 7.73 min. The ratio of occluded mice showed a significant difference (4/8 for WT mice vs 2/7 for mTOR^−/−^ mice), especially within the first 40 minutes (3/8 for WT mice vs 0/7 for mTOR^−/−^ mice). (b) For higher extent injury: The median occlusion time for WT mice (n = 12) was 21.97 ± 4.21 min, while that of mTOR^−/−^ mice (n = 11) was 13.65 ± 4.22 min. The ratios of occluded mice were 8/12 vs 9/11, for WT and mTOR^−/−^ mice, respectively.


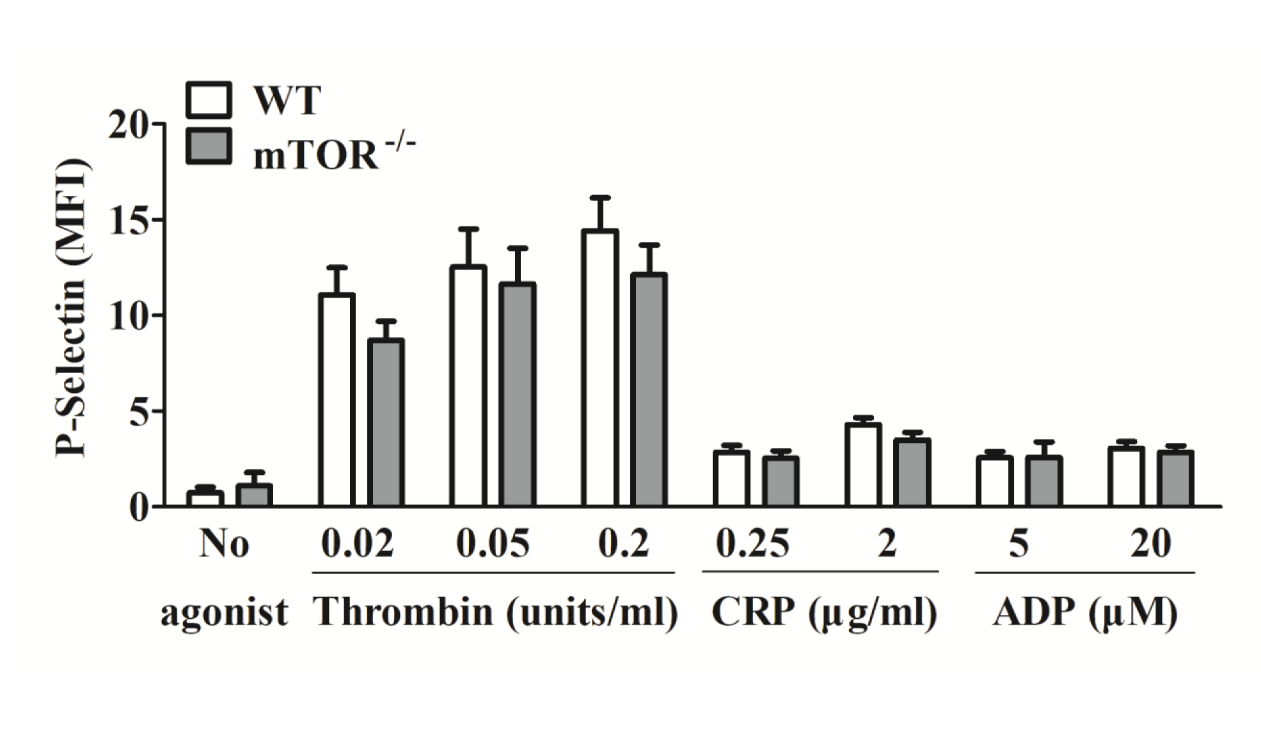


**Figure S3.** **mTOR^−/−^ platelets display** **normal α-granule secretion after** **activation with** **thrombin, collagen/CRP, or ADP.**

Washed platelets were pre-incubated with FITC-conjugated rat anti-mouse P-selectin antibody. Then, they were challenged with thrombin, collagen/CRP, or ADP. The mean fluorescence intensity (MFI) was measured by flow cytometry. Results are expressed as MFI ± SEM (n ≥ 3). The data were analyzed for statistical significance using the Student’s *t* test (*P < 0.05, **P < 0.01).





**Figure S4. Spreading on an immobilized ﬁbrinogen or collagen-coated surface is enhanced in mTOR^−/−^ platelets.**

(a) Washed platelets from WT and mTOR^−/−^ mice were pre-incubated with CaCl_2_ in the absence or presence of 0.01 U/mL thrombin for 5 minutes at 37°C (it was indicated when presence of thrombin). Then, the platelets were allowed to adhere to BSA, 50 μg/mL fibrinogen (as well as 10 μg/mL (data not shown)), 20 μg/mL collagen (as well as 5μg/mL and 50 μg/mL (data not shown)). The platelets were finally fixed, permeabilized, and stained with rhodamine phallodine. Images are representative of 3 independent experiments. The bottom images show the representative bigger or smaller-sized platelets that adhered to 20 μg/mL collagen (2╳ magnified). (b, c, d) Data from at least three independent experiments were quantified and expressed as means ± SEM (*P < 0.05, **P < 0.01, unpaired Student’s *t* test).


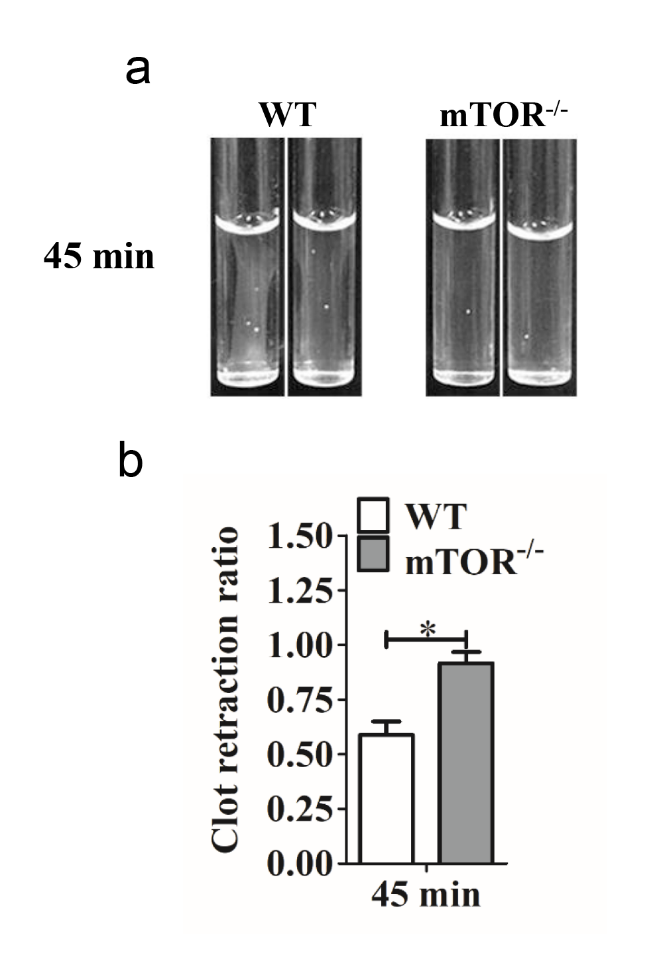


**Figure S5.** **Clot retraction in mTOR^−/−^ platelets was delayed.**

(a) Typical photograph of clot retraction at 45 min. WT or mTOR-deficient platelets were resuspended in Tyrode’s buffer and then were gently shaken after addition of 0.2 U/mL thrombin, 1 mM CaCl_2_ and 400 µg/mL soluble fibrinogen. (b) Data from at least three independent experiments were quantified and expressed as: final clot area/initial clot area. Data were expressed as means ± SEM (*P < 0.05, **P < 0.01, Paired Student’s *t* test).


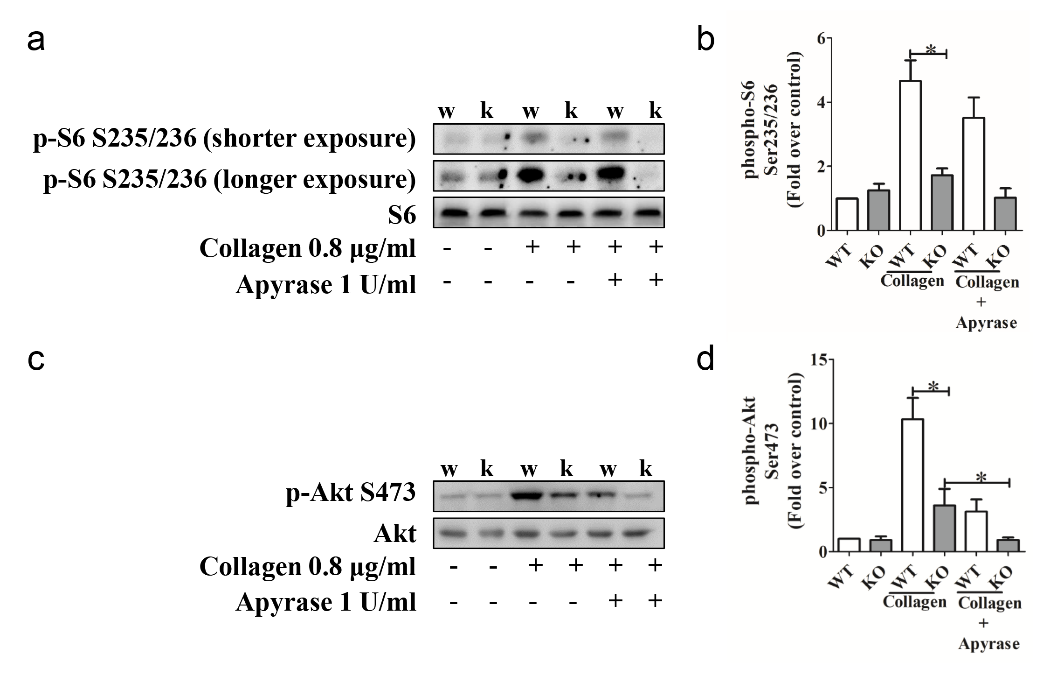


**Figure S6. Apyrase decreases the phosphorylation of S6 and Akt Ser473 in mTOR^−/−^ platelets after induction with low-concentration collagen.**

Platelets from WT (W) or mTOR^−/−^ (KO, K) mice were pre-incubated with apyrase (0 or 1 U/mL) for 5 minutes and then stimulated with collagen at the indicated low concentration under aggregating conditions for 8 minutes. (a–b, c–d) Lysates of platelets were immunoblotted with antibodies to phospho-S6 Ser235/236 and S6, phospho-Akt Ser473 and Akt. These images (separated by horizontal white space) were cropped from the different/same gels and full-length/original blots were shown in the Additional file 3: Part III. Phosphoprotein levels were normalized to S6 levels for panel b and to Akt levels for panel d. Relative values were standardized to 1 in unstimulated WT samples and represent means ± SEM from at least three independent experiments (*P < 0.05, **P < 0.01; paired Student’s *t* test).





**Figure S7. ADP rescues the phosphorylation of S6/Akt Ser473 in mTOR^−/−^ platelets when induced with low-concentration** **collagen.**

Platelets from WT (W) or mTOR^−/−^ (KO, K) mice were stimulated with collagen at the indicated low concentration under aggregating conditions for 8 minutes in the absence (buffer) or presence of ADP (10 μM), or pre-incubated with DMSO or Torin1 (500 nM) for 15 minutes. Lysates of platelets were immunoblotted with antibodies against (a, b) phospho-S6 Ser235/236 and Actin (as well as S6), (c, d) phospho-Akt Ser473 and Actin (as well as Akt), (e, f) phospho-PKCδ Thr505 and PKCδ, and (g, h) phospho-PKCε Ser729 and PKCε; these images (separated by Roman-number/horizontal-white-space) were cropped from the different/same gels and full-length/original blots were shown in the Additional file 3: Part III. Phosphoprotein levels were normalized to Actin (sometimes to S6) levels for panel b, to Actin (sometimes to Akt) levels for panel d, to PKCδ levels for panel f, and to PKCε levels for panel h. Relative values were standardized to 1 in unstimulated WT samples and represent means ± SEM from at least three independent experiments (*P < 0.05, **P < 0.01; Paired Student’s *t* test).





**Figure S8. The PKCδ-specific TAT peptide inhibitor restores the aggregation and dense granule secretion (ATP release) of mTOR***^−/−^* **platelets in response to low-dose collagen.**

Washed platelets were pre-incubated with 1 μM (δV1-1)TAT (δV1-1 conjugated to TAT for cell delivery), 5 μM (εV1-2)TAT (εV1-2 conjugated to TAT for cell delivery), TAT carrier peptide or saline (data not shown) as vehicle control for 18 minutes and then stimulated with collagen (0.5 μg/mL). (a-c) The PKCδ inhibitor (δV1-1)TAT rescued the aggregation and simultaneous ATP release of mTOR^−/−^ platelets in response to low-dose collagen (0.5 μg/mL), (d-f) while the PKCε inhibitor (εV1-2)TAT only minimally rescued the impaired aggregation and simultaneous ATP release of mTOR^−/−^ platelets in response to low-dose collagen. Data from at least three independent experiments were quantified and expressed as means ± SEM (*P < 0.05, **P < 0.01, paired Student’s *t* test). The arrows show “aggregation” or “ATP Release,” indicating 90% light transmission or 70% ATP release, respectively.

**

** **Figure S9.** **Summary of mTOR function in platelets** **and our hypothesis of** **interaction between PI3K, mTOR, PKCs and MAPKs (Erk) when stimulated by a low-dose GPVI(PAR4)-agonist.**

a (i) Based on our results and general knowledge of platelet aggregation/dense granule secretion (ATP release), activation of α_IIb_β_3_, adhesion to collagen [[13-15](#_ENREF_13)], spreading of fibrinogen and clot retraction, we summarize the mTOR function in platelets activation. a (ii) Based on our results in regards to PKCs in low-dose GPVI/PAR4-agonist-induced mTOR^−/−^ platelets (PAR4 agonist data not shown) , both existing literature regarding the role of PKCs in studies of GPVI or PARs pathways[[16-28](#_ENREF_16)], the regulation of Erk in platelet activation after stimulation of GPVI/PARs with agonists[[13](#_ENREF_13)], the regulation of PKCs by mTORC2 in other cell types[[12](#_ENREF_12)], and the regulation of Erk by mTORC2 in other cell types[[29-31](#_ENREF_29)], we hypothesize that an interaction between PI3K, mTORC2, PKCs and Erk occurs when platelets are stimulated by a low-dose GPVI(PAR4)-agonist.

**References**

1. Kucheryavykh LY, Dávilarodríguez J, Riveraaponte DE, Zueva LV, Washington AV, Sanabria P, Inyushin MY: **Platelets are responsible for the accumulation of β-amyloid in blood clots inside and around blood vessels in mouse brain after thrombosis.** *Brain Research Bulletin* 2016, **128**.

2. Stolla M, Stefanini L, Roden RC, Chavez M, Hirsch J, Greene T, Ouellette TD, Maloney SF, Diamond SL, Poncz M, et al: **The kinetics of αIIbβ3 activation determines the size and stability of thrombi in mice: implications for antiplatelet therapy.** *Blood* 2011, **117:**1005-1013.

3. Magwenzi SG, Ajjan RA, Standeven KF, Parapia LA, Naseem KM: **Factor XIII supports platelet activation and enhances thrombus formation by matrix proteins under flow conditions.** *J Thromb Haemost* 2011, **9:**820-833.

4. Au - Van Aelst B, Au - Feys HB, Au - Devloo R, Au - Vandekerckhove P, Au - Compernolle V: **Microfluidic Flow Chambers Using Reconstituted Blood to Model Hemostasis and Platelet Transfusion In Vitro.** *JoVE* 2016**:**e53823.

5. Gao C, Boylan B, Fang J, Wilcox DA, Newman DK, Newman PJ: **Heparin promotes platelet responsiveness by potentiating alphaIIbbeta3-mediated outside-in signaling.** *Blood* 2011, **117:**4946-4952.

6. Boylan B, Gao C, Rathore V, Gill JC, Newman DK, Newman PJ: **Identification of FcgammaRIIa as the ITAM-bearing receptor mediating alphaIIbbeta3 outside-in integrin signaling in human platelets.** *Blood* 2008, **112:**2780-2786.

7. Flevaris P, Stojanovic A, Gong H, Chishti A, Welch E, Du X: **A molecular switch that controls cell spreading and retraction.** *J Cell Biol* 2007, **179:**553-565.

8. Gong H, Shen B, Flevaris P, Chow C, Lam SC, Voyno-Yasenetskaya TA, Kozasa T, Du X: **G protein subunit Galpha13 binds to integrin alphaIIbbeta3 and mediates integrin "outside-in" signaling.** *Science* 2010, **327:**340-343.

9. Suzuki-Inoue K, Yatomi Y, Asazuma N, Kainoh M, Tanaka T, Satoh K, Ozaki Y: **Rac, a small guanosine triphosphate-binding protein, and p21-activated kinase are activated during platelet spreading on collagen-coated surfaces: roles of integrin alpha(2)beta(1).** *Blood* 2001, **98:**3708.

10. Torti M, Manganaro D, Visconte C, Zarà M, Canino J, Vismara M, Canobbio I, Guidetti GF: **Stimulation of mTORC2 by integrin αIIbβ3 is required for PI3Kβ-dependent activation of Akt but is dispensable for platelet spreading on fibrinogen.** *Platelets* 2019**:**1-9.

11. Shen B, Zhao X, O'Brien KA, Stojanovic-Terpo A, Delaney MK, Kim K, Cho J, Lam SC, Du X: **A directional switch of integrin signalling and a new anti-thrombotic strategy.** *Nature* 2013, **503:**131-135.

12. Laplante M, Sabatini DM: **mTOR signaling in growth control and disease.** *Cell* 2012, **149:**274-293.

13. Li Z, Delaney MK, O'Brien KA, Du X: **Signaling During Platelet Adhesion and Activation.** *Arterioscler Thromb Vasc Biol* 2010, **30:**2341-2349.

14. Nieswandt B, Watson SP: **Platelet-collagen interaction: is GPVI the central receptor?** *Blood* 2003, **102:**449-461.

15. Varga-Szabo D, Pleines I, Nieswandt B: **Cell Adhesion Mechanisms in Platelets.** *Arterioscler Thromb Vasc Biol* 2008, **28:**403-412.

16. Gilio K, Harper MT, Cosemans JM, Konopatskaya O, Munnix IC, Prinzen L, Leitges M, Liu Q, Molkentin JD, Heemskerk JW, Poole AW: **Functional divergence of platelet protein kinase C (PKC) isoforms in thrombus formation on collagen.** *J Biol Chem* 2010, **285:**23410-23419.

17. Harper MT, Poole AW: **Diverse functions of protein kinase C isoforms in platelet activation and thrombus formation.** *J Thromb Haemost* 2010, **8:**454-462.

18. Pula G, Schuh K, Nakayama K, Nakayama KI, Walter U, Poole AW: **PKCdelta regulates collagen-induced platelet aggregation through inhibition of VASP-mediated filopodia formation.** *Blood* 2006, **108:**4035-4044.

19. Chari R, Getz T, Nagy B, Jr., Bhavaraju K, Mao Y, Bynagari YS, Murugappan S, Nakayama K, Kunapuli SP: **Protein kinase C[delta] differentially regulates platelet functional responses.** *Arterioscler Thromb Vasc Biol* 2009, **29:**699-705.

20. Hall KJ, Harper MT, Gilio K, Cosemans JM, Heemskerk JW, Poole AW: **Genetic analysis of the role of protein kinase Ctheta in platelet function and thrombus formation.** *PLoS One* 2008, **3:**e3277.

21. Poole MTHaAW: **PKCtheta in platelet activation.** *blood* 2009, **114:** 489-491.

22. Nagy B, Bhavaraju K, Getz T, Bynagari YS, Kim S, Kunapuli SP: **Impaired activation of platelets lacking protein kinase C-θ isoform.** *Blood* 2009, **113:**2557-2567.

23. Bynagari-Settipalli YS, Lakhani P, Jin J, Bhavaraju K, Rico MC, Kim S, Woulfe D, Kunapuli SP: **Protein kinase C isoform epsilon negatively regulates ADP-induced calcium mobilization and thromboxane generation in platelets.** *Arterioscler Thromb Vasc Biol* 2012, **32:**1211-1219.

24. Pears CJ, Thornber K, Auger JM, Hughes CE, Grygielska B, Protty MB, Pearce AC, Watson SP: **Differential roles of the PKC novel isoforms, PKCdelta and PKCepsilon, in mouse and human platelets.** *PLoS One* 2008, **3:**e3793.

25. Unsworth AJ, Smith H, Gissen P, Watson SP, Pears CJ: **Submaximal Inhibition of Protein Kinase C Restores ADP-induced Dense Granule Secretion in Platelets in the Presence of Ca2+.** *Journal of Biological Chemistry* 2011, **286:**21073-21082.

26. Cohen S, Braiman A, Shubinsky G, Ohayon A, Altman A, Isakov N: **PKCtheta is required for hemostasis and positive regulation of thrombin-induced platelet aggregation and alpha-granule secretion.** *Biochem Biophys Res Commun* 2009, **385:**22-27.

27. Konopatskaya O, Gilio K, Harper MT, Zhao Y, Cosemans JM, Karim ZA, Whiteheart SW, Molkentin JD, Verkade P, Watson SP, et al: **PKCalpha regulates platelet granule secretion and thrombus formation in mice.** *J Clin Invest* 2009, **119:**399-407.

28. Swaminathan Murugappan FT, Robert T.Dorsam, Haripriya Shankar,, Kunapuli aSP: **Differential role of protein kinase C delta isoform in agonist-induced dense granule secretion in human platelets.** *J Biol Chem* 2004, **279** 2360-2367.

29. Chen XG, Liu F Fau - Song X-F, Song Xf Fau - Wang Z-H, Wang Zh Fau - Dong Z-Q, Dong Zq Fau - Hu Z-Q, Hu Zq Fau - Lan R-Z, Lan Rz Fau - Guan W, Guan W Fau - Zhou T-G, Zhou Tg Fau - Xu X-M, Xu Xm Fau - Lei H, et al: **Rapamycin regulates Akt and ERK phosphorylation through mTORC1 and mTORC2 signaling pathways.** 2010.

30. Edwards BS, Isom WJ, Navratil AM: **Gonadotropin releasing hormone activation of the mTORC2/Rictor complex regulates actin remodeling and ERK activity in LbetaT2 cells.** 2016.

31. Jindra PT, Jin Yp Fau - Jacamo R, Jacamo R Fau - Rozengurt E, Rozengurt E Fau - Reed EF, Reed EF: **MHC class I and integrin ligation induce ERK activation via an mTORC2-dependent pathway.** 2008.
