# Supplementary material for: mTOR regulates GPVI-mediated platelet activation
Source: J Transl Med. 2021 May 10;19:201. doi: 10.1186/s12967-021-02756-y (PMC8111939; doi:10.1186/s12967-021-02756-y)
Supplement: Supplementary file 2 — Additional file 2. Additional Information Part I [file 12967_2021_2756_MOESM2_ESM.docx]

**Additional file 2: Additional Information Part I**

**mTOR regulates GPVI-mediated platelet activation**

This document provides detailed summary diagrams for interpretating the Additional file 1: Figure S9.

**
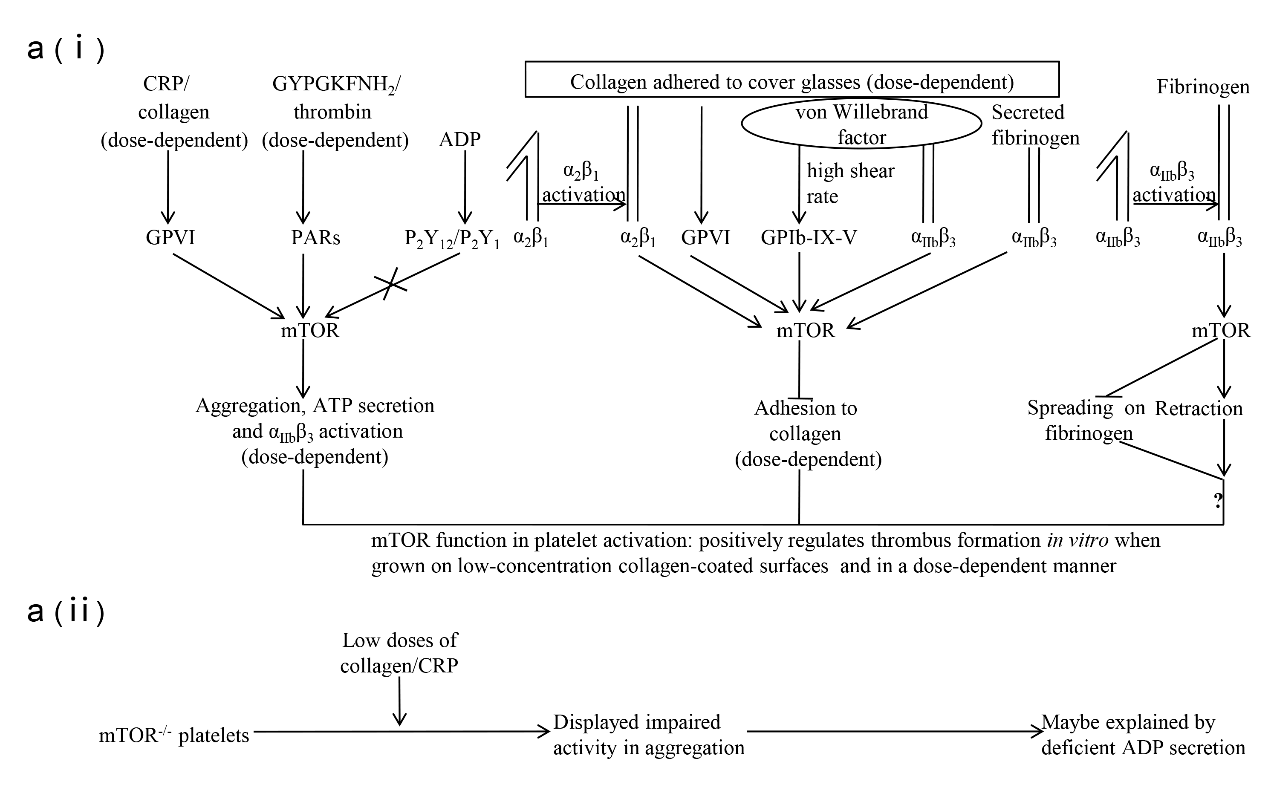
**

**Additional Information 1.** **Summary of mTOR function in platelets.**

a (i) and a (ii) Based on our results and general knowledge of platelet aggregation, dense granule secretion (ATP release, part of data not shown), activation of α_IIb_β_3_, adhesion to collagen [[1-3](#_ENREF_1)], spreading of fibrinogen and clot retraction, we summarize the mTOR function in platelets activation.





**Additional Information 2.** **Hypothesized interaction between mTOR and α_IIb_β_3_.**

b (i), b (ii), b (iii) Based on previous work [[4-7](#_ENREF_4)], we hypothesize an interaction between mTOR and α_IIb_β3.


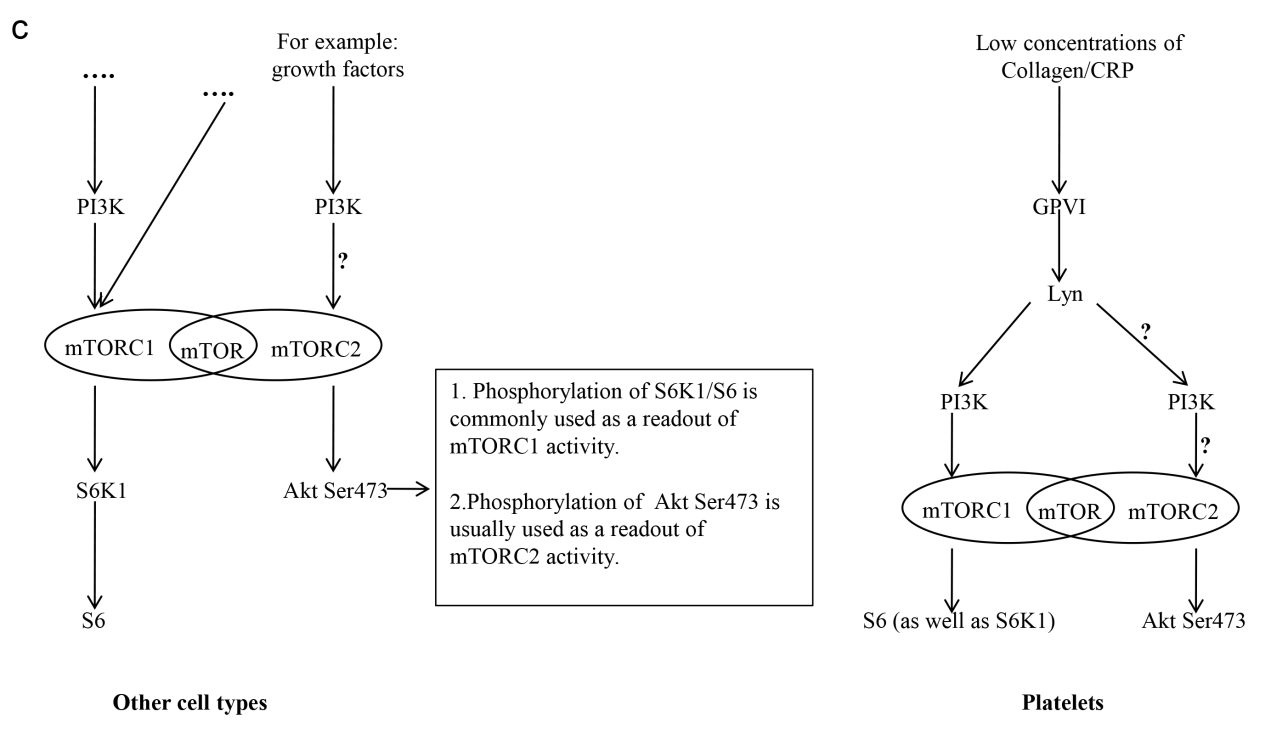


**Additional Information 3.** **The impaired downstream signaling of mTOR in mTOR^-/-^ platelets.**

**(c)** Activation of S6K1 and phosphorylation of S6 and Akt Ser473 are usually used as indicators of mTORC1 and mTORC2 activity, respectively[[7](#_ENREF_7), [8](#_ENREF_8)]. Additionally, consistent with[[9-12](#_ENREF_9)], the phosphorylation of S6 was used as the sole readout of mTORC1 activity in our study. Thus, we confirmed the deficiency in low-dose GPVI agonist-induced mTOR signaling by the impaired phosphorylation of S6 and Akt Ser473, despite that phosphorylation of these molecules was unablated when pre-incubated without apyrase (in usually).





**Additional Information 4.** **Hypothesized the reason why phosphorylation of the substrates in mTOR^-/-^ platelets was not typically ablated.**

d (i) Interaction between Erk and S6K1/S6 in other cell types [[13-18](#_ENREF_13)]. d (ii) The phosphorylation level of S6 and Akt Ser473 in mTOR*^−/−^* platelets was decreased by the exogenous apyrase when induction with low concentrations of collagen or PAR4 agonist (data not shown). d (iii) Based on d (i) and d (ii), we hypothesize that, the ADP that was secreted from the dense granule of mTOR^-/-^ platelets when activated by GPVI/PAR4 agonists, can activated P_2_Ys and bypassed mTOR complexes, and finally phosphorylated Akt Ser473 or S6 Ser235/236 (possibly through ERK [[13-18](#_ENREF_13)]) in mTOR^-/-^ platelets. These phosphorylation events can be amplified by an ADP secretion cascade. This may be the primary, or even the sole reason, why deletion of mTOR did not typically result in ablation of S6/Akt Ser473 phosphorylation in the absence of exogenous apyrase.





**Additional Information 5. Hypothesized interaction between PI3K, mTOR,**

**PKCs and MAPKs (Erk) when stimulated by a low-dose GPVI(PAR4)-agonist.**

e (i) PKCs have been also been reported to be regulated by mTOR or mTORC2 in other cell types[[7](#_ENREF_7)]. e (ii) Our results demonstrate that mTOR regulates PKCδ/ε, especially PKCδ but not PKCα/β/θ in platelets after activation by a low concentration of GPVI-dependent agonist. e (iii) The PKC δ inhibitor rottlerin or (δV1-1)TAT, but not the PKCα/β inhibitor Go6976, restored the aggregation of mTOR^-/-^ platelets in response to low-dose collagen, while the PKCε inhibitor (εV1-2)TAT only minimally rescued the impaired aggregation of mTOR^-/-^ platelets in response to low-dose collagen. e (iv) and e( v) Based on e (ii),e (iii), and our results in regards to Erk (data not shown), as well as the existing literature regarding the role of PKCs in studies of GPVI or PARs pathways [[19-31](#_ENREF_19)], the regulation of Erk in platelet activation after stimulation of GPVI/PARs with agonists [[1](#_ENREF_1)], the regulation of PKCs by mTORC2 in other cell types [[7](#_ENREF_7)], the regulation of Erk by mTORC2 in other cell types [[32-34](#_ENREF_32)], we hypothesize that an interaction between PI3K, mTORC2, PKCs and Erk occurs when platelets are stimulated by a low-dose GPVI(PAR4)-agonist.

***References***

1. Li Z, Delaney MK, O'Brien KA, Du X: **Signaling During Platelet Adhesion and Activation.** *Arterioscler Thromb Vasc Biol* 2010, **30:**2341-2349.

2. Nieswandt B, Watson SP: **Platelet-collagen interaction: is GPVI the central receptor?** *Blood* 2003, **102:**449-461.

3. Varga-Szabo D, Pleines I, Nieswandt B: **Cell Adhesion Mechanisms in Platelets.** *Arterioscler Thromb Vasc Biol* 2008, **28:**403-412.

4. Flevaris P, Stojanovic A, Gong H, Chishti A, Welch E, Du X: **A molecular switch that controls cell spreading and retraction.** *J Cell Biol* 2007, **179:**553-565.

5. Gong H, Shen B, Flevaris P, Chow C, Lam SC, Voyno-Yasenetskaya TA, Kozasa T, Du X: **G protein subunit Galpha13 binds to integrin alphaIIbbeta3 and mediates integrin "outside-in" signaling.** *Science* 2010, **327:**340-343.

6. Shen B, Zhao X, O'Brien KA, Stojanovic-Terpo A, Delaney MK, Kim K, Cho J, Lam SC, Du X: **A directional switch of integrin signalling and a new anti-thrombotic strategy.** *Nature* 2013, **503:**131-135.

7. Laplante M, Sabatini DM: **mTOR signaling in growth control and disease.** *Cell* 2012, **149:**274-293.

8. Hong S, Inoki K: **Evaluating the mTOR Pathway in Physiological and Pharmacological Settings.** *Methods Enzymol* 2016, **587:**405-428.

9. Mori H, Inoki K, Münzberg H, Opland D, Faouzi M, Villanueva EC, Ikenoue T, Kwiatkowski D, MacDougald OA, Jr. MGM: **Critical Role for Hypothalamic mTOR Activity in Energy Balance.** *Cell Metabolism* 2009, **9:**362-374.

10. Wang J, Zhang C-J, Zhang J, He Y, Lee YM, Chen S, Lim TK, Ng S, Shen H-M, Lin Q: **Mapping sites of aspirin-induced acetylations in live cells by quantitative acid-cleavable activity-based protein profiling (QA-ABPP).** *Sci Rep* 2015, **5:**7896.

11. Wang J, Zhang J, Lee Y-M, Koh P-L, Ng S, Bao F, Lin Q, Shen H-M: **Quantitative chemical proteomics profiling of de novo protein synthesis during starvation-mediated autophagy.** *Autophagy* 2016, **12:**1931-1944.

12. Menon S, Yecies JL, Hui HZ, Howell JJ, Nicholatos J, Harputlugil E, Bronson RT, Kwiatkowski DJ, Manning BD: **Chronic Activation of mTOR Complex 1 Is Sufficient to Cause Hepatocellular Carcinoma in Mice.** *Science Signaling* 2011, **5:**ra24.

13. Dennis PB, Pullen N, Pearson RB, Kozma SC, Thomas G: **Phosphorylation sites in the autoinhibitory domain participate in p70(s6k) activation loop phosphorylation.** *J Biol Chem* 1998, **273:**14845-14852.

14. Gangarossa G, Perroy J, Valjent E: **Combinatorial topography and cell-type specific regulation of the ERK pathway by dopaminergic agonists in the mouse striatum.** *Brain Struct Funct* 2013, **218:**405-419.

15. Gobert D, Topolnik L, Azzi M, Huang L, Badeaux F, Desgroseillers L, Sossin WS, Lacaille JC: **Forskolin induction of late-LTP and up-regulation of 5' TOP mRNAs translation via mTOR, ERK, and PI3K in hippocampal pyramidal cells.** *J Neurochem* 2008, **106:**1160-1174.

16. Mukhopadhyay NK, Price DJ, Kyriakis JM, Pelech S, Sanghera J, Avruch J: **An array of insulin-activated, proline-directed serine/threonine protein kinases phosphorylate the p70 S6 kinase.** *J Biol Chem* 1992, **267:**3325-3335.

17. Pullen, N.: **Phosphorylation and Activation of p70s6k by PDK1.** *Science* 1998, **279:**707-710.

18. Roux PP, Shahbazian D, Vu H, Holz MK, Cohen MS, Taunton J, Sonenberg N, Blenis J: **RAS/ERK signaling promotes site-specific ribosomal protein S6 phosphorylation via RSK and stimulates cap-dependent translation.** *J Biol Chem* 2007, **282:**14056-14064.

19. Gilio K, Harper MT, Cosemans JM, Konopatskaya O, Munnix IC, Prinzen L, Leitges M, Liu Q, Molkentin JD, Heemskerk JW, Poole AW: **Functional divergence of platelet protein kinase C (PKC) isoforms in thrombus formation on collagen.** *J Biol Chem* 2010, **285:**23410-23419.

20. Harper MT, Poole AW: **Diverse functions of protein kinase C isoforms in platelet activation and thrombus formation.** *J Thromb Haemost* 2010, **8:**454-462.

21. Pula G, Schuh K, Nakayama K, Nakayama KI, Walter U, Poole AW: **PKCdelta regulates collagen-induced platelet aggregation through inhibition of VASP-mediated filopodia formation.** *Blood* 2006, **108:**4035-4044.

22. Chari R, Getz T, Nagy B, Jr., Bhavaraju K, Mao Y, Bynagari YS, Murugappan S, Nakayama K, Kunapuli SP: **Protein kinase C[delta] differentially regulates platelet functional responses.** *Arterioscler Thromb Vasc Biol* 2009, **29:**699-705.

23. Hall KJ, Harper MT, Gilio K, Cosemans JM, Heemskerk JW, Poole AW: **Genetic analysis of the role of protein kinase Ctheta in platelet function and thrombus formation.** *PLoS One* 2008, **3:**e3277.

24. Poole MTHaAW: **PKCtheta in platelet activation.** *blood* 2009, **114:** 489-491.

25. Nagy B, Bhavaraju K, Getz T, Bynagari YS, Kim S, Kunapuli SP: **Impaired activation of platelets lacking protein kinase C-θ isoform.** *Blood* 2009, **113:**2557-2567.

26. Bynagari-Settipalli YS, Lakhani P, Jin J, Bhavaraju K, Rico MC, Kim S, Woulfe D, Kunapuli SP: **Protein kinase C isoform epsilon negatively regulates ADP-induced calcium mobilization and thromboxane generation in platelets.** *Arterioscler Thromb Vasc Biol* 2012, **32:**1211-1219.

27. Pears CJ, Thornber K, Auger JM, Hughes CE, Grygielska B, Protty MB, Pearce AC, Watson SP: **Differential roles of the PKC novel isoforms, PKCdelta and PKCepsilon, in mouse and human platelets.** *PLoS One* 2008, **3:**e3793.

28. Unsworth AJ, Smith H, Gissen P, Watson SP, Pears CJ: **Submaximal Inhibition of Protein Kinase C Restores ADP-induced Dense Granule Secretion in Platelets in the Presence of Ca2+.** *Journal of Biological Chemistry* 2011, **286:**21073-21082.

29. Cohen S, Braiman A, Shubinsky G, Ohayon A, Altman A, Isakov N: **PKCtheta is required for hemostasis and positive regulation of thrombin-induced platelet aggregation and alpha-granule secretion.** *Biochem Biophys Res Commun* 2009, **385:**22-27.

30. Konopatskaya O, Gilio K, Harper MT, Zhao Y, Cosemans JM, Karim ZA, Whiteheart SW, Molkentin JD, Verkade P, Watson SP, et al: **PKCalpha regulates platelet granule secretion and thrombus formation in mice.** *J Clin Invest* 2009, **119:**399-407.

31. Swaminathan Murugappan FT, Robert T.Dorsam, Haripriya Shankar,, Kunapuli aSP: **Differential role of protein kinase C delta isoform in agonist-induced dense granule secretion in human platelets.** *J Biol Chem* 2004, **279** 2360-2367.

32. Chen XG, Liu F Fau - Song X-F, Song Xf Fau - Wang Z-H, Wang Zh Fau - Dong Z-Q, Dong Zq Fau - Hu Z-Q, Hu Zq Fau - Lan R-Z, Lan Rz Fau - Guan W, Guan W Fau - Zhou T-G, Zhou Tg Fau - Xu X-M, Xu Xm Fau - Lei H, et al: **Rapamycin regulates Akt and ERK phosphorylation through mTORC1 and mTORC2 signaling pathways.** 2010.

33. Edwards BS, Isom WJ, Navratil AM: **Gonadotropin releasing hormone activation of the mTORC2/Rictor complex regulates actin remodeling and ERK activity in LbetaT2 cells.** 2016.

34. Jindra PT, Jin Yp Fau - Jacamo R, Jacamo R Fau - Rozengurt E, Rozengurt E Fau - Reed EF, Reed EF: **MHC class I and integrin ligation induce ERK activation via an mTORC2-dependent pathway.** 2008.
