# Supplementary material for: mTOR regulates GPVI-mediated platelet activation
Source: J Transl Med. 2021 May 10;19:201. doi: 10.1186/s12967-021-02756-y (PMC8111939; doi:10.1186/s12967-021-02756-y)
Supplement: Supplementary file 3 — Additional file 3. Additional Information Part II & III [file 12967_2021_2756_MOESM3_ESM.docx]

**Additional file 3: Additional Information Part II & III**

**mTOR regulates GPVI-mediated platelet activation**

This document provides additional information with detailed “ADP can rescue the impaired dense granule secretion (ATP release) and platelet aggregation induced by low concentrations of collagen in mTOR^−/−^ platelets”, “APC-fibrinogen”, “Analysis of Lyn and Erk phosphorylation”, “ADP rescues the phosphorylation of Erk in mTOR^−/−^ platelets when induced with low-concentration collagen” data as well as Additional file 1: Figure S4, which are part or even not shown in the main text and Additional file 1 (Additional Information Part II).

This document also provides additional information with full-length/original gels/blots from figures (or additional figures) and with **v**ideos for figure 1 (d-i) or figure s1 (13-parts of videos; figure s1 refer to Additional file 1: Figure S1) and original data of flow cytometry (CD62P, JON/A, APC-fibrinogen).





**Additional Information 6. Impaired ADP secretion may explain the deficient platelet aggregation induced by low- concentration collagen in mTOR^−/−^ platelets.**

(a) The addition of ADP (10 μM) rescued the impaired aggregation of mTOR^−/−^ platelets when simulation with 0.5 μg/m collagen or/and 0.75 μg/mL CRP (as well as 0.8 μg/mL collagen (data not shown)); (b) Washed platelets from WT or mTOR^−/−^ mice were challenged with 0.5 μg/mL collagen after pre-incubation with apyrase (0 or 1 U/mL) for 5 minutes, and aggregation and ATP release were evaluated; (c–d) The percentage aggregation and levels of ATP release after stimulation with 0.5 μg/mL collagen are indicated as means ± SEM from at least three independent experiments (*P < 0.05, **P < 0.01; paired Student’s *t* test). The arrows show “aggregation” or “ATP Release,” indicating 90% light transmission or 70% ATP release, respectively.


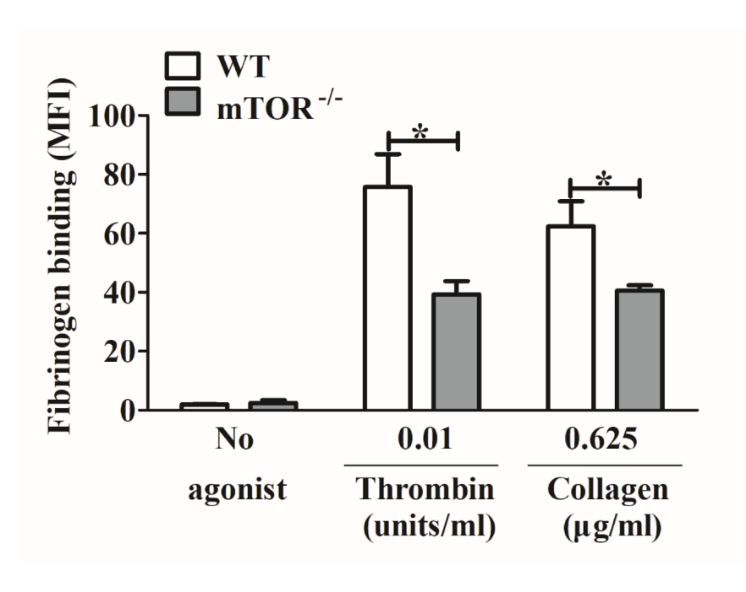


**Additional Information 7. Detailed “APC-fibrinogen” Data**

**mTOR^−/−^ platelets exhibit impaired activation of α_IIb_β_3_ after low-dose collagen or thrombin stimulation.**

Washed platelets were pre-incubated with APC- fibrinogen. Then, they were activated with thrombin or collagen at a low concentration. The mean fluorescence intensity (MFI) was measured by ﬂow cytometry. Results are expressed as MFI ± SEM (n ≥ 3). The data were analyzed for statistical significance using the Student’s *t* test (*P < 0.05, **P < 0.01).





**Additional Information 8. Detailed figure s4 (figure s4 refers to Additional file 1: Figure S4; spreading on an immobilized ﬁbrinogen or collagen-coated surface is enhanced in mTOR^−/−^ platelets).**

(a) Washed platelets from WT and mTOR^−/−^ mice were pre-incubated with CaCl_2_ in the absence or presence of 0.01 U/mL thrombin for 5 minutes at 37°C (it was indicated when presence of thrombin). Then, the platelets were allowed to adhere to BSA, 10 or 50 μg/mL fibrinogen, 5, 20 or 50 (data not shown) μg/mL collagen. The platelets were finally fixed, permeabilized, and stained with rhodamine phallodine. Images are representative of 3 independent experiments. The bottom-middle images show the representative bigger or smaller-sized platelets that adhered to 20 μg/mL collagen (2╳ magnified). (b, c, d) Data from at least three independent experiments were quantified and expressed as means ± SEM (*P < 0.05, **P < 0.01, unpaired Student’s *t* test).





**Additional Information 9. Analysis of Lyn and Erk phosphorylation**

Platelets from WT (W) or mTOR^−/−^ (KO, K) mice were stimulated with the GPVI agonist CRP or the PAR4 agonist GYPGKF-NH_2_ at the indicated low concentration for 5.5 minutes. (a, b-e) Lysates of platelets were immunoblotted with antibodies to phospho-Lyn Tyr507 and Actin, phospho–Erk Thr202/Tyr204 and Erk1/2; these images (separated by horizontal white space) were cropped from the different/same gels and full-length/original blots were shown in the Part III. Phosphoprotein levels were normalized to Erk1/2 levels for panels c. The phosphoprotein levels (upper band, U) were normalized to Erk1/2 levels for panels e, however, the lower band (L) was done at more exposure time. Relative values were standardized to 1 in unstimulated WT samples and represent means ± SEM from at least three independent experiments (*P < 0.05, **P < 0.01; Paired Student’s *t* test).


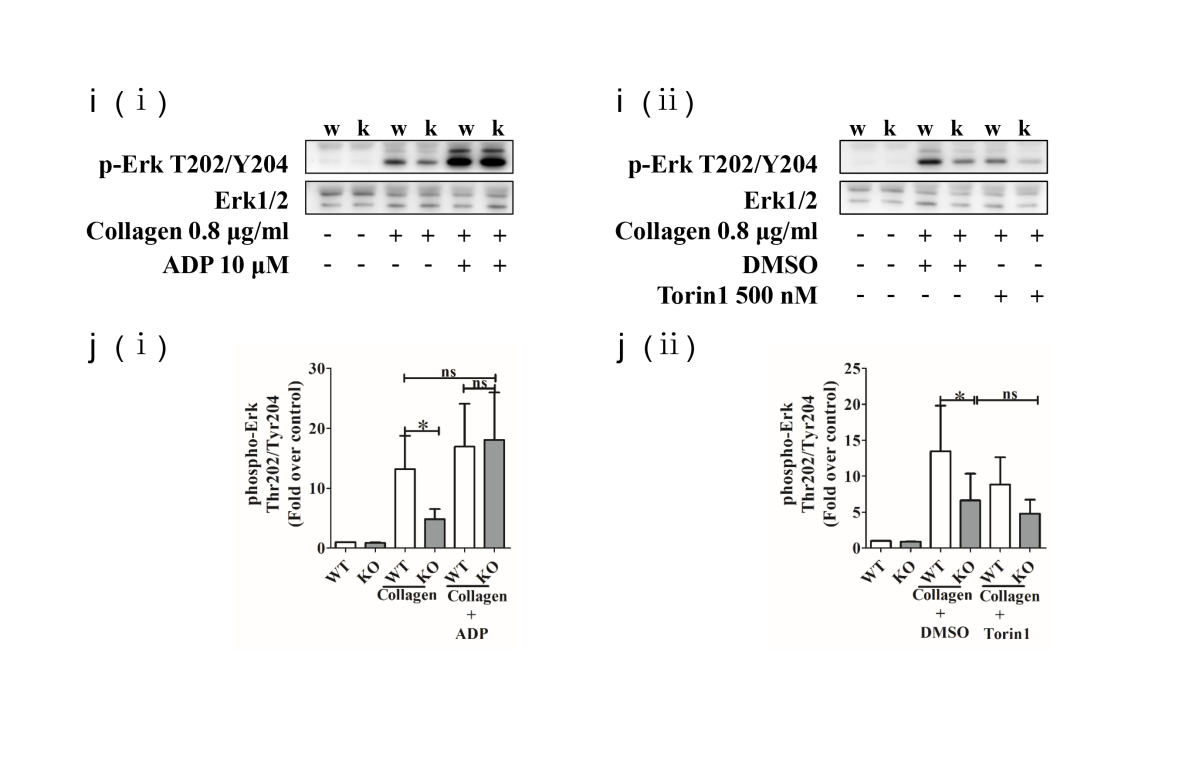


**Additional Information 10. ADP rescues the phosphorylation of Erk in mTOR^−/−^ platelets when induced with low-concentration** **collagen.**

Platelets from WT (W) or mTOR^−/−^ (KO, K) mice were stimulated with collagen at the indicated low concentration under aggregating conditions for 8 minutes in the absence (buffer) or presence of ADP (10 μM), or pre-incubated with DMSO or Torin1 (500 nM) for 15 minutes. Lysates of platelets were immunoblotted with antibodies against (i, j) phospho–Erk Thr202/Tyr204 and Erk1/2; these images (separated by Roman-number/horizontal-white-space) were cropped from the different/same gels and full-length/original blots were shown in the Part III. Phosphoprotein levels were normalized to Erk1/2 levels for panel j. Relative values were standardized to 1 in unstimulated WT samples and represent means ± SEM from at least three independent experiments (*P < 0.05, **P < 0.01; Paired Student’s *t* test).


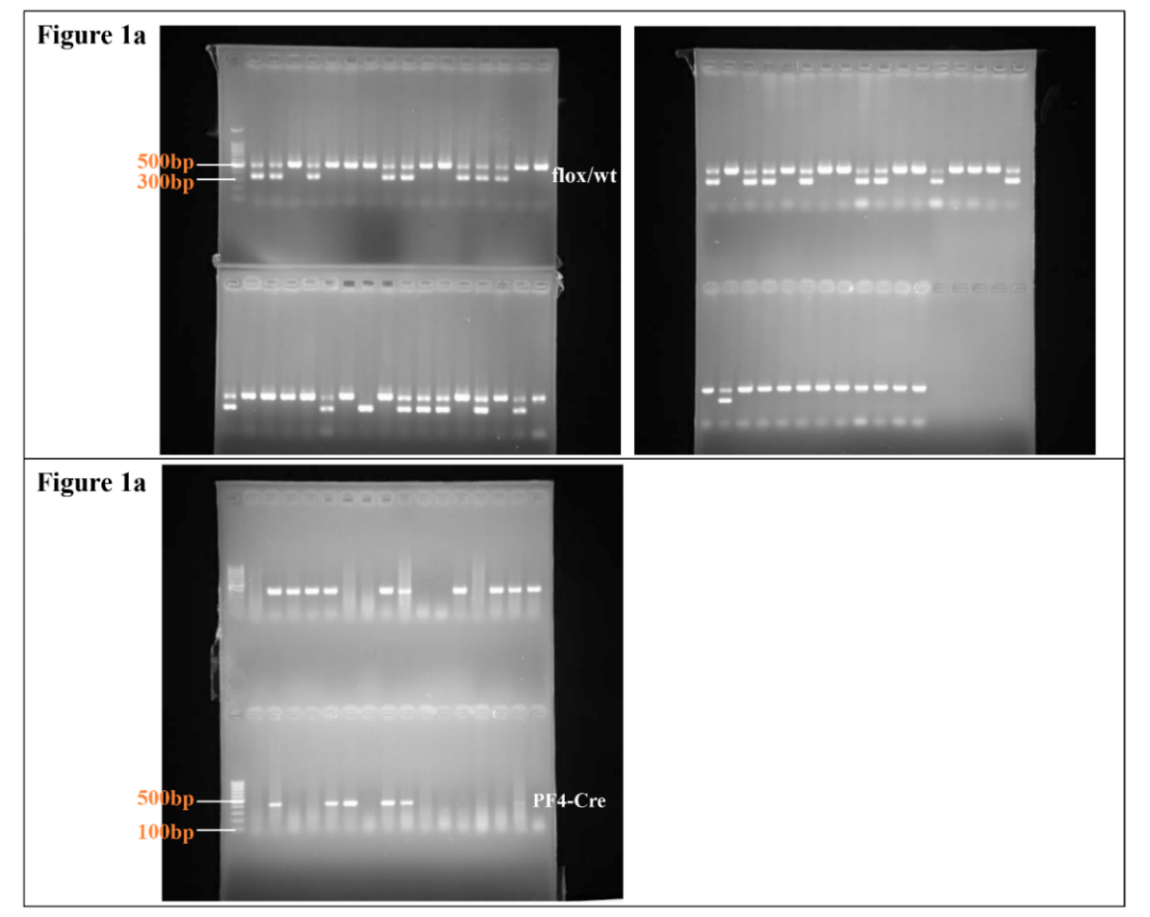


**
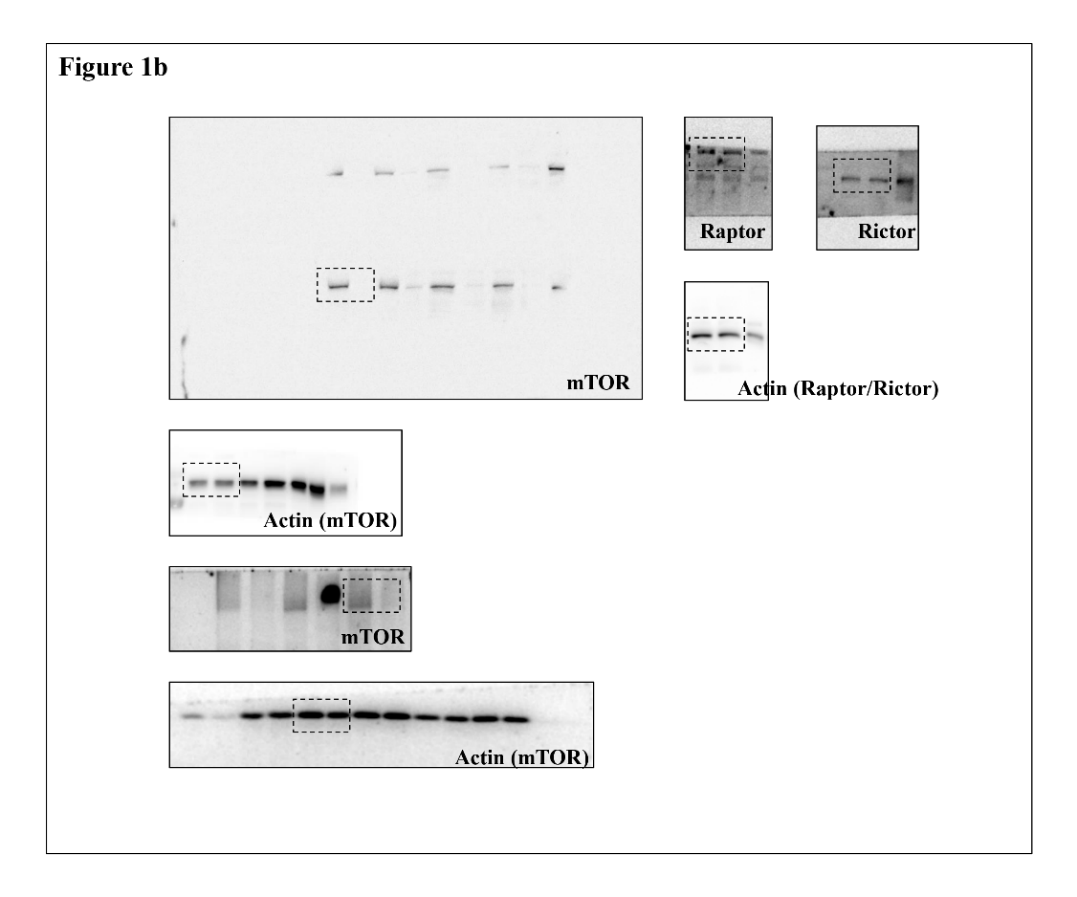
**
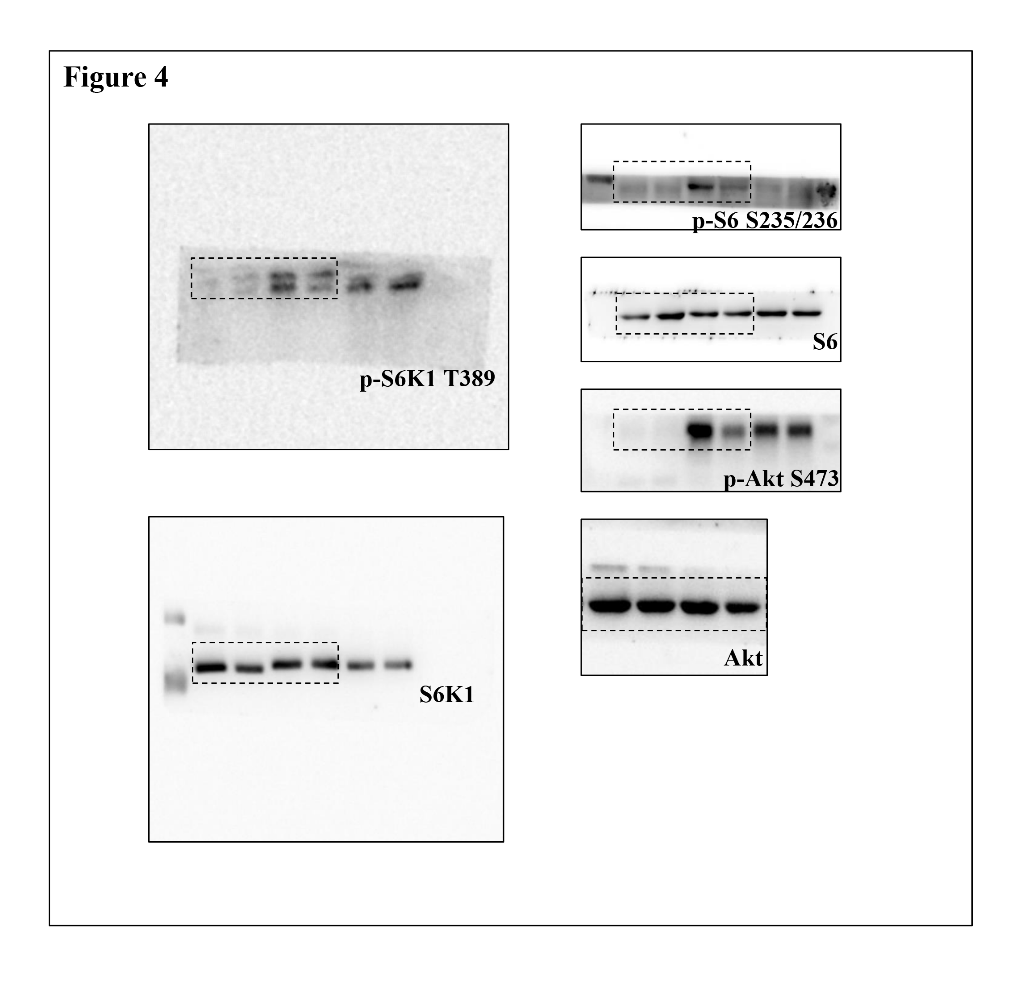


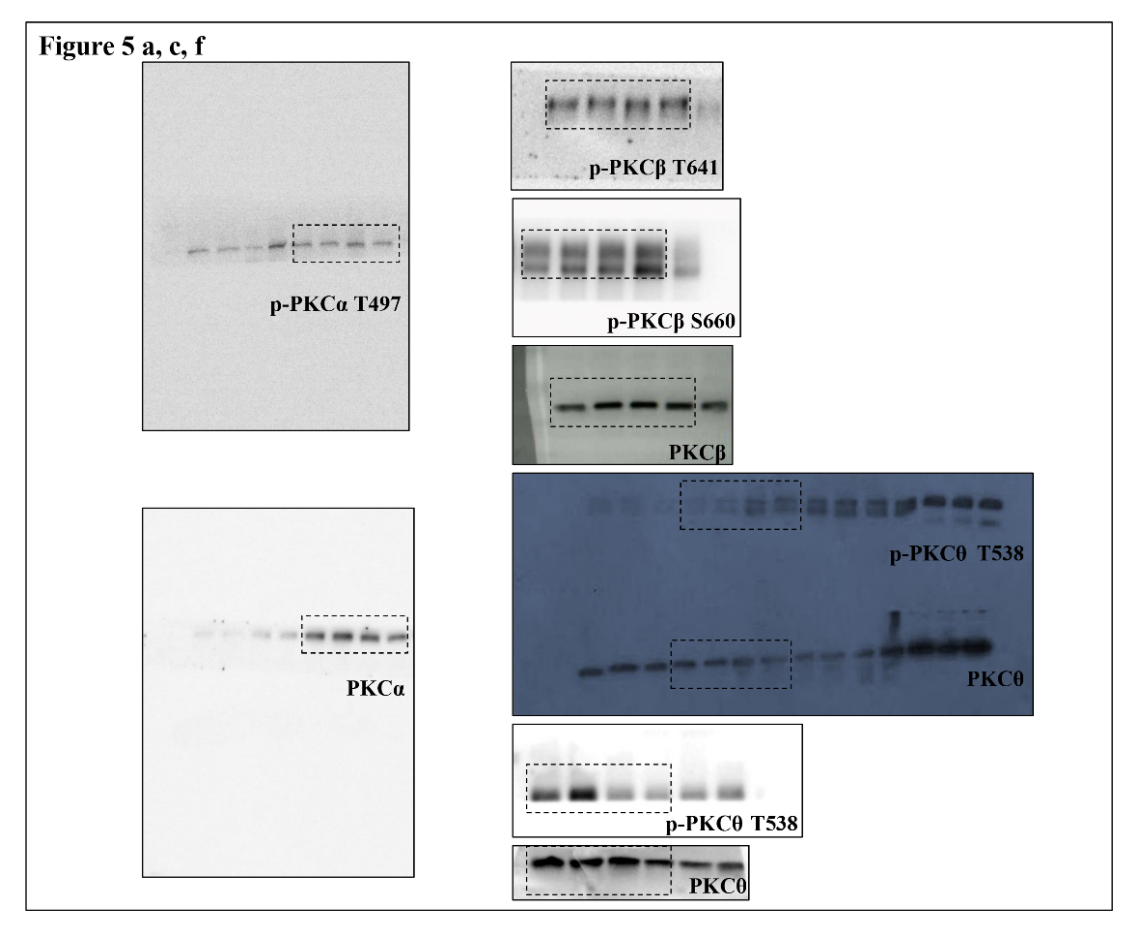


**
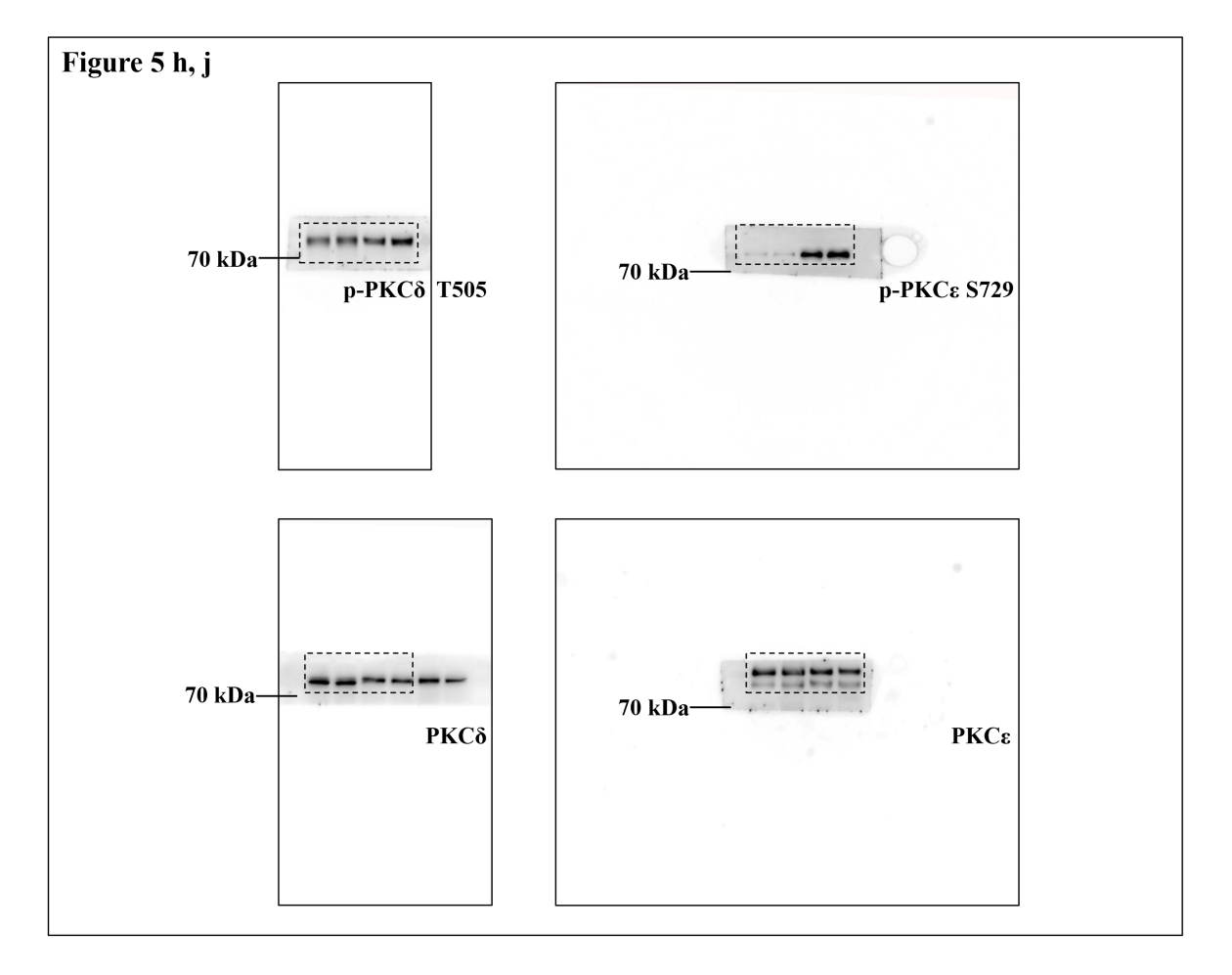
**

**
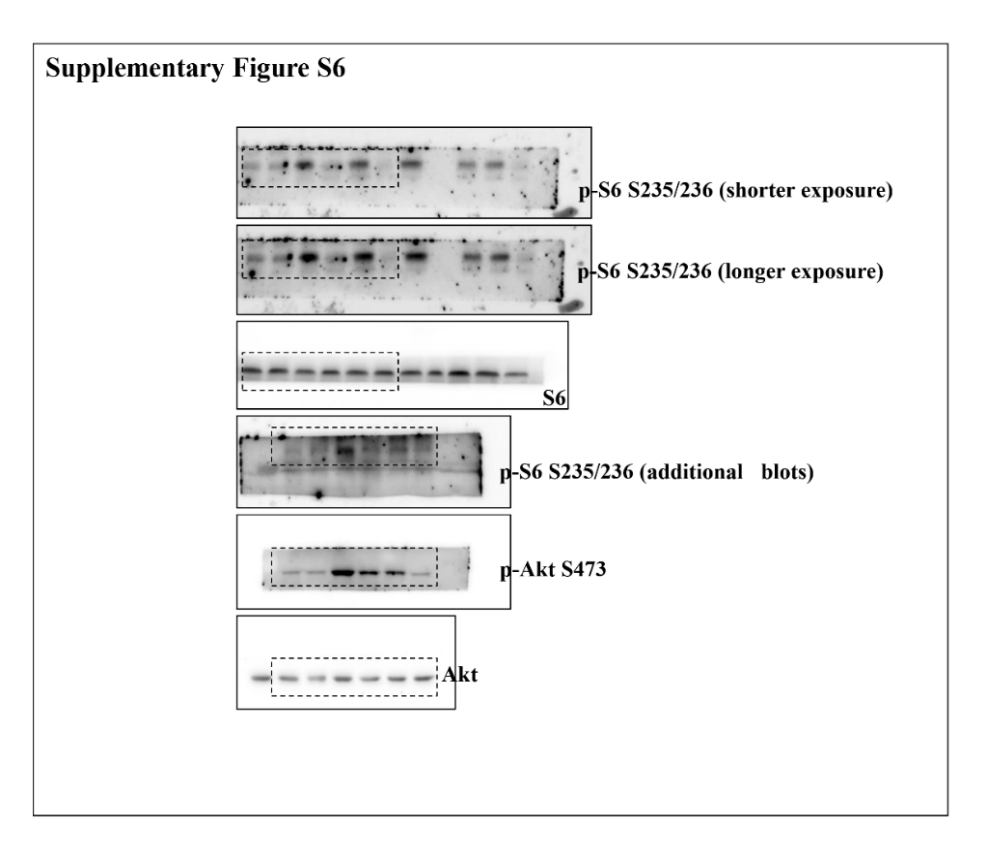
**

**
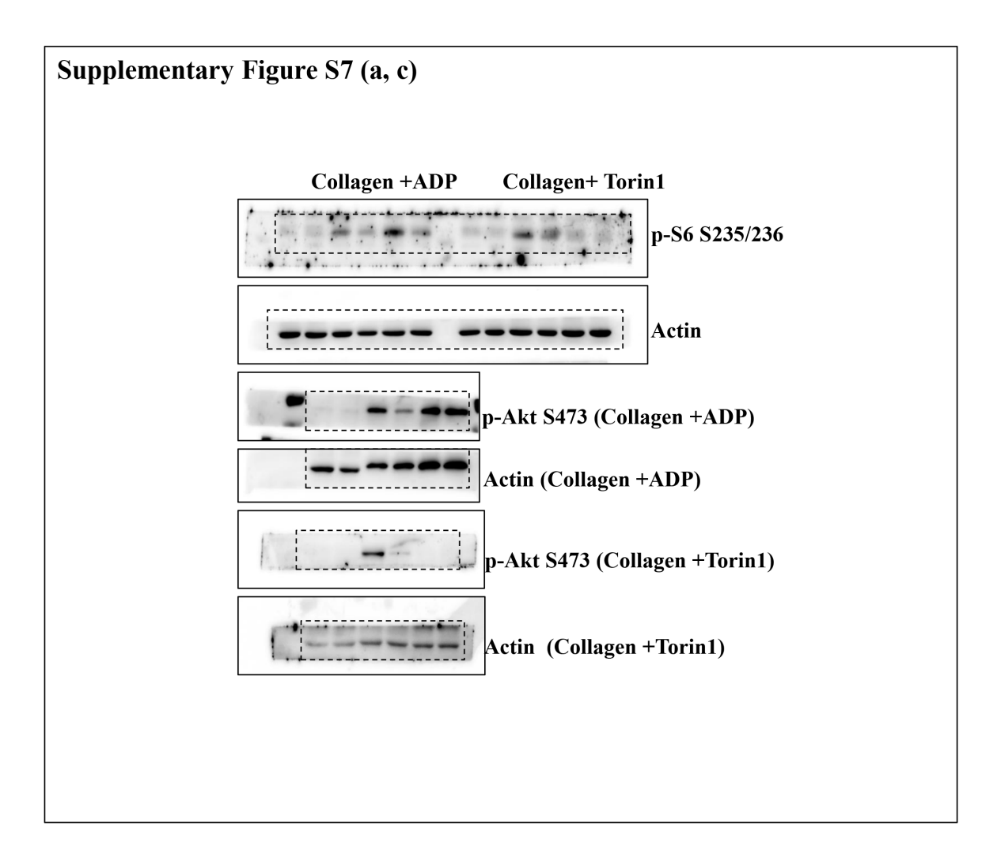
**

**
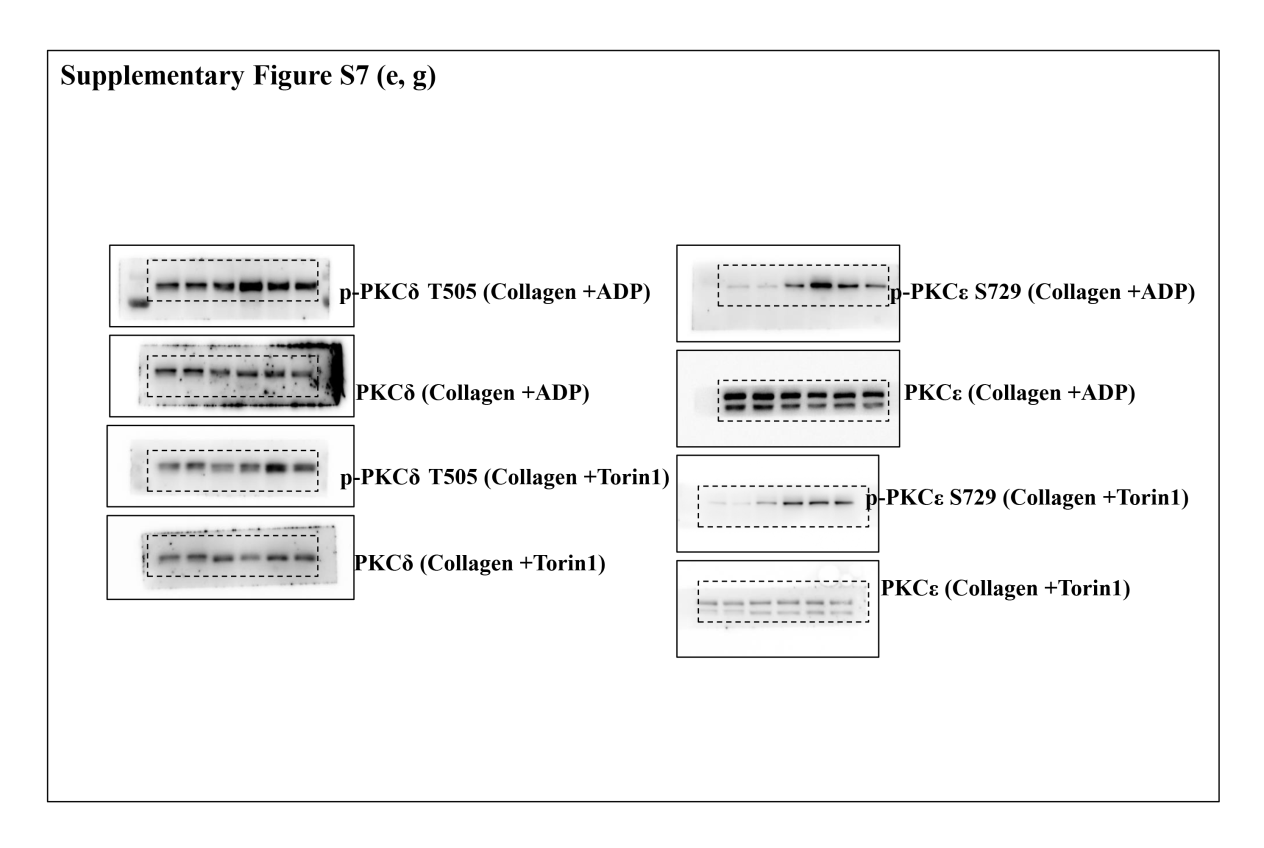
**

**
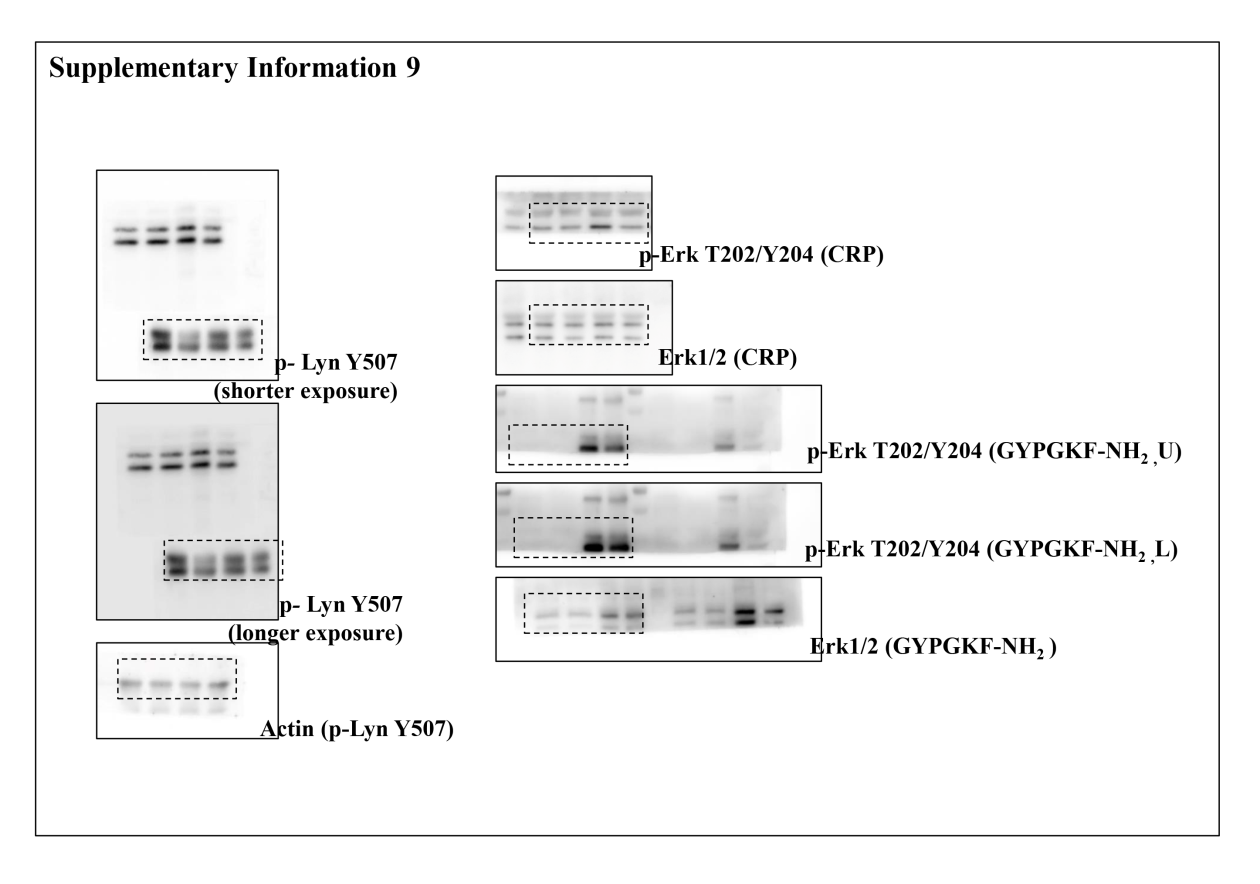
**

**
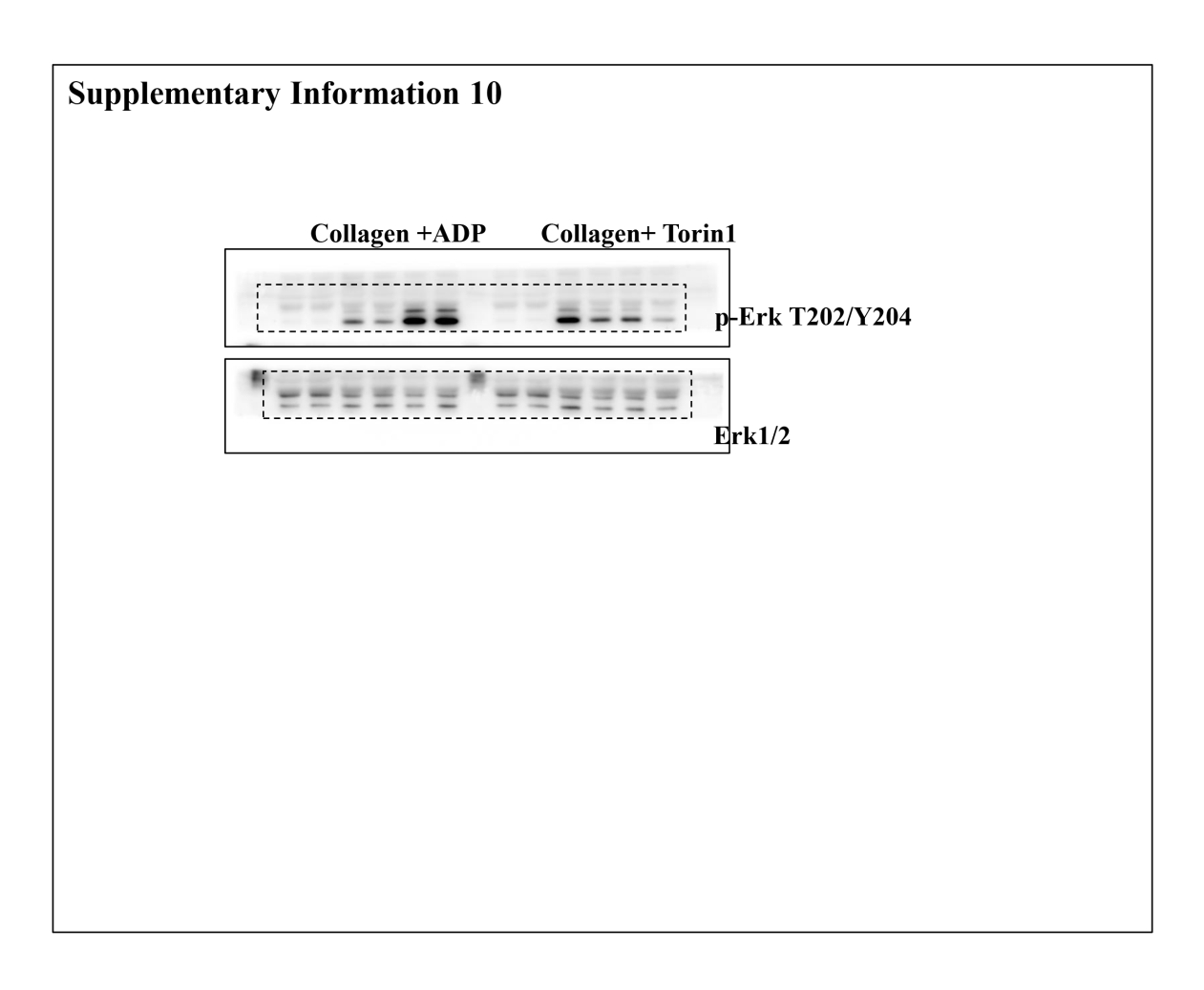
**

**Additional Information 11.** **The full-length gels or blots from figure 1 (a, b), figure 4, figure 5, figure s6, figure s7, additional information 9 and additional information 10**

Full-length/original gels or blots corresponding to the figures (1(a, b), 4 and 5), additional figures (s6-7; Additional file 1: Figure S6-7) and additional information (9-10; Additional file 3: Additional Information 9-10). The full-length gels corresponding to figure 1a show genotyping results that similar to the cropped section in figure 1a. In addition to the full-length/original blots in figure 1b, figure 5f, we also provide additional groups of full-length/original blots of mTOR and Actin, phospho-PKCθ Thr538 and PKCθ. These results are the same/similar as the cropped section in figure 1b, figure 5f, but the Actin/ PKCθ are more smooth. In addition to the full-length/original blots in additional figure s6, we also provide additional full-length/original blots of phospho-S6 Ser235/236, these results are the same as the cropped section in additional figure s6-a. Dashed lines show the cropped section displayed on figures (1(a, b), 4 and 5) or additional figures (s6-7) or additional information (9-10) or show the same/similar evidences as those. In both, the images show other bands corresponding to the some other different experimental conditions.

**Additional Information 12. Videos for figure 1 (d-i) or figure s1 (13-parts of videos) and original data of flow cytometry (for figure 3, figure s3 or additional information 7 (Additional file 3: Additional Information 7))**

We provide videos (parts of) for figure 1or figure s1 (Additional file 1: Figure S1) and the original data of flow cytometry (CD62P, JON/A, APC-fibrinogen):

Videos (7-pairs of) of using whole blood (or videos (6-pairs of) of using recombinant whole blood, the results were shown in figure 1 or figure s1) for perfusion on low/high concentration collagen (for whole blood: 4 times for low concentration collagen and 3 times for higher concentration collagen; for recombinant whole blood: 3 times for low concentration collagen and 3 times for higher concentration collagen) were provided in the URL. In these videos, the whole (or reconstituted) blood from mTOR^−/−^ mice showed impaired thrombus formation when perfused in lower concentration (20 μg/mL) collagen-coated flow chambers, and the whole (or reconstituted) blood from mTOR^−/−^ mice also showed overcame the defective thrombus formation when perfused in higher concentration (50 μg/mL) collagen-coated surfaced.  Some videos displayed perfusion and observation of  WT/mTOR^−/−^  at same time in 10 ╳ objective lens, and others videos displayed perfusion and observation of WT or mTOR^−/−^  independently. The titles of the videos and folders identify the genotype, collagen concentration, blood or reconstituted blood, and orders of the experiments, etc. The perfusion were lasted for 5 minutes, and all videos were recorded before the beginning of perfusion, and most videos were taken longer than corresponding perfusion time. In the “50ug collagen_ reconstituted blood_ 2nd” folder, additional images and video were also provided to clarify the results.

We also upload the folder of the original data of flow cytometry: mTOR^−/−^platelets showed impaired activation of α_IIb_β_3_after stimulation with a low dose of thrombin or collagen washed platelets after incubation with anti α_IIb_β_3_ were not affected by mTOR deficiency when stimulated at higher concentrations (the results were shown in Figure 3). However, the expression of P-selectin was not influenced by mTOR deficiency (the results were shown in Additional file 1: Figure S3). Some raw data of independent experiments are provided in folders in the URL (for P-selectin: we have uploaded only two experimental results (while the original data of ADP-related expression of P-selectin was provided four results), because we think these two results can explain the expression of P-selectin was not objective by mTOR deficiency). In the JON/A experiment, the concentration of the lowest thrombin in the first experiment and the second experiment are not the same. Because we were still trying to find out about the low-concentration thrombin, the collagen may have some similar situation. However, when there were labeled significant changes between WT and KO in the figure, we provided at least three times of the original data of the stimulant concentration used. Moreover, we believe that the results of other concentrations are consistent with the trend shown in the figure.

Please click the following link to view the videos that for figure 1 or figure s1 (13-parts of videos) and to view the original data of flow cytometry: <https://pan.baidu.com/s/1TXiX6r>

NFsEEvtCTEKC5F4A and please enter the code: hrdt for visiting the folder.
